# H3K9me selectively blocks transcription factor activity and ensures differentiated tissue integrity

**DOI:** 10.1038/s41556-021-00776-w

**Published:** 2021-11-04

**Authors:** Stephen P. Methot, Jan Padeken, Giovanna Brancati, Peter Zeller, Colin E. Delaney, Dimos Gaidatzis, Hubertus Kohler, Alexander van Oudenaarden, Helge Großhans, Susan M. Gasser

**Affiliations:** 1 Friedrich Miescher Institute for Biomedical Research, Basel, Switzerland; 2 Hubrecht Institute-KNAW and University Medical Center, Utrecht, The Netherlands; 3 Oncode Institute, Utrecht, The Netherlands; 4 Swiss Institute of Bioinformatics, Basel, Switzerland; 5 Faculty of Natural Sciences, University of Basel, Basel, Switzerland; 6 Present Address: Department of Biosystems Science and Engineering, ETH Zurich, Basel, Switzerland

**Keywords:** Histone post-translational modifications, Nuclear organization, Transcriptional regulatory elements, Differentiation

## Abstract

The developmental role of histone H3K9 methylation (H3K9me), which typifies heterochromatin, remains unclear. In *Caenorhabditis elegans*, loss of H3K9me leads to a highly divergent upregulation of genes with tissue and developmental-stage specificity. During development H3K9me is lost from differentiated cell type-specific genes and gained at genes expressed in earlier developmental stages or other tissues. The continuous deposition of H3K9me2 by the SETDB1 homolog MET-2 after terminal differentiation is necessary to maintain repression. In differentiated tissues, H3K9me ensures silencing by restricting the activity of a defined set of transcription factors at promoters and enhancers. Increased chromatin accessibility following the loss of H3K9me is neither sufficient nor necessary to drive transcription. Increased ATAC-seq signal and gene expression correlate at a subset of loci positioned away from the nuclear envelope, while derepressed genes at the nuclear periphery remain poorly accessible despite being transcribed. In conclusion, H3K9me deposition can confer tissue-specific gene expression and maintain the integrity of terminally differentiated muscle by restricting transcription factor activity.

## Abstract (web summary)

Gasser and colleagues report that H3K9me2 and H3K9me3 repress lineage-specific and germline genes by restricting the activity of a subset of transcription factors in terminally differentiated *Caenorhabditis* *elegans* tissues.

## Main

Development in multicellular organisms is governed by a carefully orchestrated programme of gene expression that is controlled by both genetic and epigenetic factors^1,2^. Lysine 9 methylation on histone H3 (H3K9me) is a defining modification of heterochromatin. In multicellular eukaryotes, heterochromatin serves two main functions: it silences the transcription of satellite repeats and transposons^3^ and it silences tissue-specific genes during development^4,5^. Consistent with this, the loss of appropriately targeted heterochromatin is associated with a loss of tissue integrity, ageing and cancer^4–8^.

Silencing of repetitive elements (REs) prevents the accumulation of repeat-RNA-driven RNA:DNA hybrids, which in turn leads to replication stress, genome instability and infertility^3,9^. In contrast, the importance of H3K9me-mediated repression at genes is less clear. In mice, the inhibition of H3K9-specific histone methyl transferases (HMTs) boosts the efficiency of pluripotent-stem-cell induction^10,11^, and the deletion of H3K9me3 HMT SUV39H1/H2, coupled with an endoderm-specific deletion of SETDB1 (ref. ^4^) or conditional deletion of G9a^12^, compromises tissue integrity^4^. These findings suggest developmental roles for H3K9me, yet interpretations are complicated by the partial redundancy among the many mammalian H3K9 HMTs and their roles in chromosome segregation. In contrast, *Caenorhabditis elegans* has only two somatic H3K9 HMTs, and mutant worms that lack all detectable H3K9me have been generated^13^. Surprisingly, these animals develop from embryos to adults^3,14,15^, allowing one to examine how H3K9me controls gene expression in differentiated tissues.

We examined the tissue-specific expression and accessibility of H3K9me-marked sequences following the loss of MET-2, a SETDB1-like HMT responsible for H3K9me1 and H3K9me2 (refs. ^13,16^), and SET-25, a G9a/SUV39H-like enzyme mediating H3K9me3 (refs. ^13,17,18^). Although H3K9me2 is indeed necessary for silencing, gene activation in its absence requires a defined set of transcription factors (TFs). Surprisingly, whereas H3K9me restricts TF binding, the loss of H3K9me does not necessarily result in chromatin decompaction and enhanced sensitivity in the Assay for Transposase-Accessible (Tn5) Chromatin by high-throughput sequencing (ATAC-seq). Subnuclear localization with respect to the nuclear envelope may influence whether or not transcription and accessibility correlate.

## Gene derepression following H3K9me loss is tissue specific

**Fig. 1:**
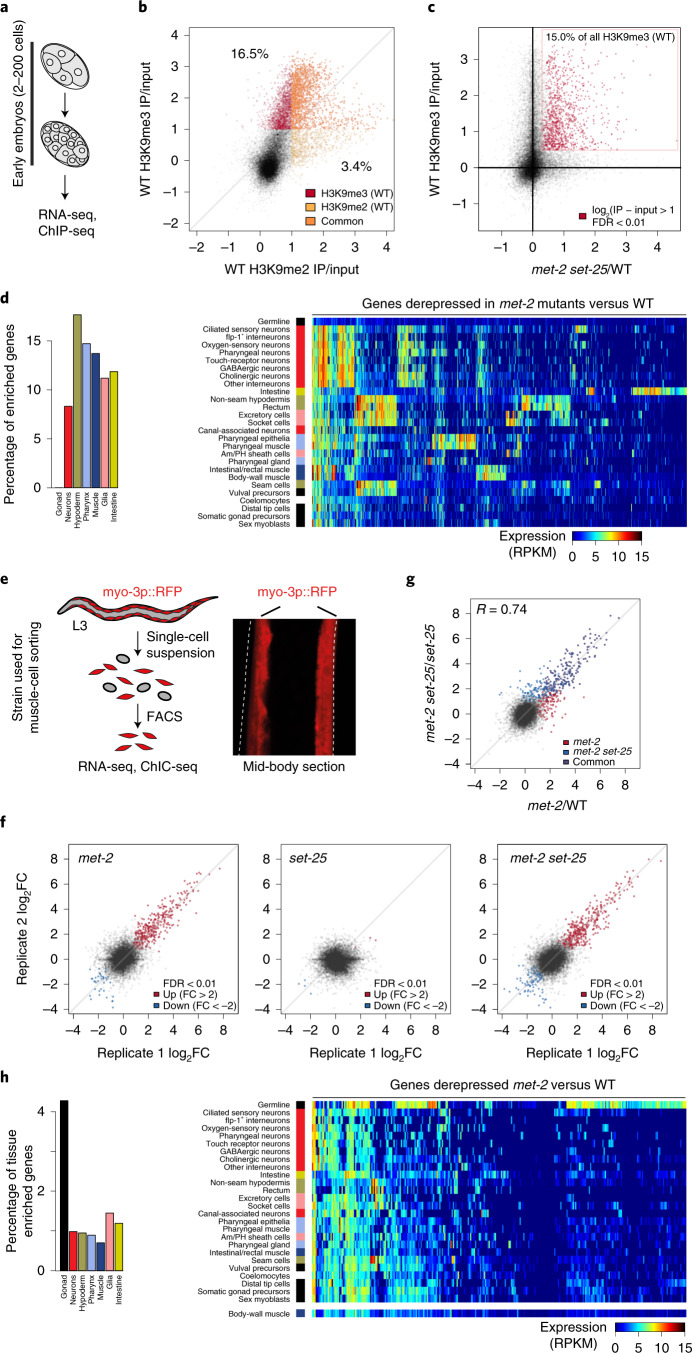
Limited derepression of H3K9me3-marked tissue-specific genes following the loss of H3K9me2/me3. **a**, Isolation of early embryos for RNA-seq and ChIP–seq. **b**, H3K9me2 and H3K9me3 on genes mapped by ChIP–seq in early embryos at 20 °C. Correlation between the mean H3K9me3 and mean H3K9me2 at genes (log_2_FC values normalized to the input). Individual genes are coloured based on enrichment for H3K9me2 (3.4%), or H3K9me3 and Common (16.5%) or neither (black); FC > 2; *n* = 2 independent biological replicates. **c**, Correlation between the mean H3K9me3 (as in **a**) and the mean log_2_-transformed FC in gene expression in the *met-2 set-25* double mutant normalized to the WT (*n* = 3 independent biological replicates). Individual genes (red) are both enriched for H3K9me3 in WT (FC > 2) and derepressed in the *met-2 set-25* double-mutant embryos (FDR < 0.01). **b**,**c**, IP, immunoprecipitation. **d**, The expression of genes that are derepressed in *met-2-*mutant embryos is restricted to specific tissues in WT L2 larvae^20^. Percentage of genes that are derepressed in *met-2-*mutant embryos (*n* = 3 independent biological replicates) over the total number of tissue-specific genes for each tissue (left; number of genes: gonad, 0; neurons, 313; hypoderm, 302; pharynx, 247; muscle, 180; glia, 204; and intestine, 313). Heatmap of the expression levels of each derepressed gene (columns) in individual cell types (rows; the coloured bar on the left indicates their parent tissue) in WT L2 larvae. The tissue and cell-type expression data are from^20^. **e**, Isolation of muscle cells for RNA-seq and ChIC-seq from L3 larvae by FACS of cells expressing myo-3p::RFP (left). Image of L3 larva with muscle-specific RFP expression (right). **f**, Gene expression (normalized to the WT) in sorted muscle cells of the indicated mutants (two biologically independent replicates are shown). Genes identified as significantly changed are shown in colour. **g**, Correlation between the gene expression (log_2_FC) between the *met-2* and *met-2 set-25* double mutants. Significantly changed loci (FDR < 0.01 and FC > 2) are coloured according to the genotype and those that changed in both genotypes are shown in dark blue. **h**, Genes that are derepressed in *met-2* muscle (*n* = 2 independent biological replicates) as a percentage of all tissue-specific genes in WT L2 larvae (left; gene number: gonad, 172; neurons, 30; hypoderm, 25; pharynx, 13; muscle, 7; glia, 33; and intestine, 28) and as a heatmap of derepressed genes (columns) according to the cell type (right).

**Extended Data Fig. 1:**
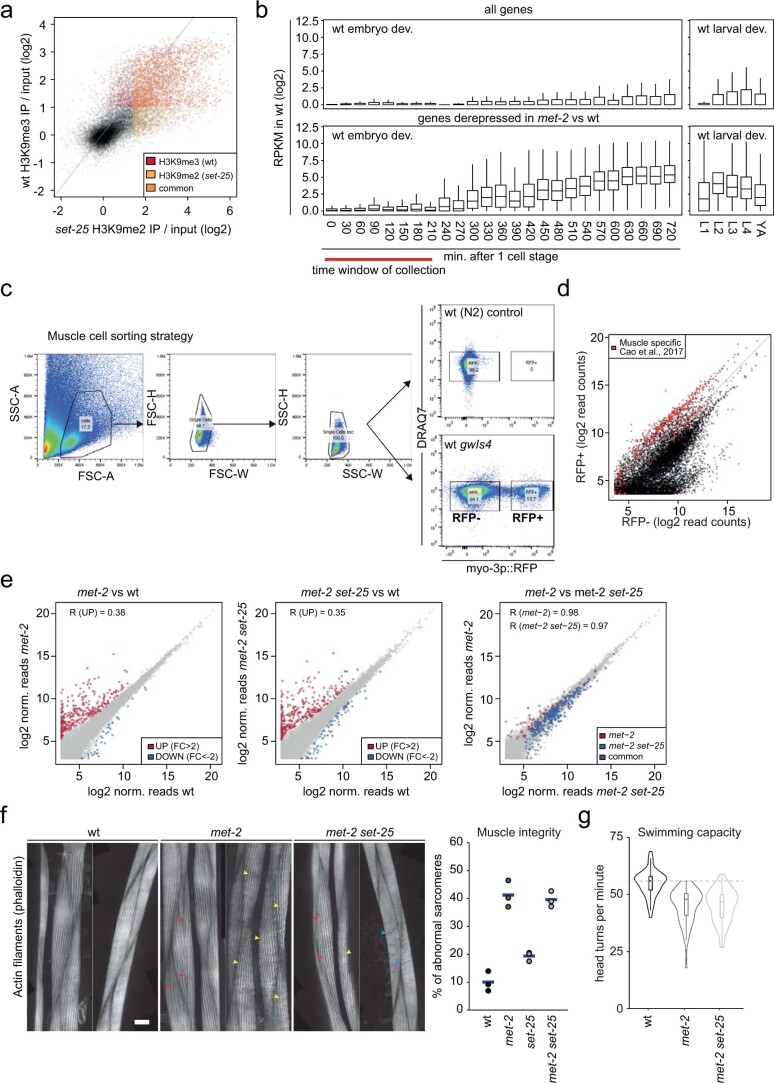
Muscle integrity is compromised in the absence of H3K9me2/me3. a) Scatter plot correlating mean log2FC of H3K9me3 over input at genes in wt embryos to the mean log2FC of H3K9me2 over input at genes in *set-25* mutant embryos. Genes coloured based on enrichment for H3K9me3 (red), H3K9me2 (yellow), both (orange), or neither (black). b) The wt expression of all *C. elegans* genes (*top)* or the subset of genes derepressed in *met-2* deficient embryos (*bottom)*, plotted over embryogenic time, or distinct larval stages. Gene sets are displayed as boxplots showing median, boxes 50% and whiskers 5-95% of the group. Outliers excluded to simplify interpretation. Time window of collection refers to early embryos. c) Sorting scheme for muscle cells expressing myo-3p::RFP. Negative (wt) control was always included to ensure proper gating. Note the gating strategy for DRAQ7 negative (viability marker) and RFP- (non-muscle) or RFP + (muscle cells) from wt *gwIS4* animals. d) Scatter plot showing gene expression as log2 of read counts from cells sorted as RFP + or RFP-. Individual muscle specific genes (based on scRNA-seq^1^) are highlighted in red. N = 3 biological independent experiments. e) *Left*, Scatter plots correlating the absolute gene expression of normalized read counts (log2) from wt muscle cells to *met-2* or *met-2 set-25* mutant muscle cells. Genes significantly changed in mutants (see Fig. 1f) are highlighted in colour (FDR < 0.01 and FC > 2, red or < -2, blue). N = 3 biological independent experiments. *Right*, Scatter plot, comparing absolute expression in *met-2* and *met-2 set-25* mutants. Genes significantly upregulated in mutants relative to wt are coloured according to genotype, with genes significantly changed in both mutants coloured in purple. Pearson correlation coefficients (R) calculated for genes significantly upregulated in indicated mutants vs wt. f) *Left*, representative max-projection images of muscle actin filaments stained with phalloidin-rhodamine in young adults grown at 20^o^C. Arrowheads indicate defective filaments (red, fused; yellow, wavy; blue, broken). Scale bar, 10 µm. *Right*, quantification of the proportion of sarcomeres with defective actin filaments. (N = 3 biological independent experiments). g) Swimming capacity, measured by transferring worms into liquid and counting the number of head movements per minute. Head movements are plotted as violin plots containing boxplots of median, boxes 50% and whiskers 90% of the group. Grey bar indicates median count from wt worms. Data shown represent one experiment with n = 50 worms/genotype. Assay performed four times with similar results. Source data

We set out to discover whether H3K9me alone controls gene expression in *C. elegans* and whether this depends on the developmental stage or tissue type. Genome-wide mapping of H3K9me2 and H3K9me3 (H3K9me2/me3) in early *C. elegans* embryos (up to the 200-cell stage) showed that 16.5% of all genes were marked by H3K9me3 and 3.4% were marked by H3K9me2 (Fig. 1a,b)^3^. Nonetheless, the loss of MET-2 resulted in higher and more widespread gene derepression than the loss of SET-25 (refs. ^18,19^), and in the *set-25* mutant H3K9me2 was retained on the loci that carried H3K9me3 in wild-type worms (Extended Data Fig. 1a)^18^. This indicates that MET-2-mediated H3K9me2 precedes and largely compensates for H3K9me3 in embryos^18^. Nonetheless, even following the loss of all H3K9me2/me3 in the *met-2 set-25* double mutant, only about 15% of the genes that carried H3K9me3 in wild-type (WT) embryos were derepressed (Fig. 1c).

Characterization of the genes derepressed following the loss of H3K9me2/me3 revealed that larval genes are expressed prematurely in *met-2*-deficient early embryos (Extended Data Fig. 1b). We plotted the derepressed genes according to their expression patterns in a WT tissue atlas generated from larval stage 2 (L2) single-cell transcriptomes^20^ (Fig. 1d). The genes derepressed in embryos cluster into groups of cell type-specific genes from all somatic tissues except the germline. This provides evidence that H3K9me2/me3 are needed in embryos to prevent the precocious activation of genes typically expressed at later developmental stages.

To explore H3K9me-mediated gene silencing in differentiated tissues, we optimized the fluorescence-activated cell sorting (FACS)-based isolation of differentiated cell types from a single-cell suspension derived from L3 larvae. By sorting red fluorescent protein (RFP)^+^ cells from a strain with muscle-specific RFP expression (*gwIs4*; Fig. 1e)^21^, we were able to perform RNA-seq on a highly enriched population of differentiated muscle cells (Extended Data Fig. 1c,d). A comparison of the gene expression in the H3K9 HMT mutants and the WT showed that 310 and 336 genes (false-discovery rate (FDR) < 0.01; log_2_-transformed fold change (FC), log_2_FC > 2), respectively, were upregulated in the *met-2*- and *met-2 set-25-*mutant muscle cells (Fig. 1f). Only three genes were upregulated in the *set-25* mutant and there was a strong correlation between the transcriptional changes in the *met-2*- and *met-2 set-25*-mutant cells (*met-2*/WT versus *met-2 set-25*/*set-25* log_2_FC, Pearson’s correlation coefficient (*R*) = 0.74; Fig. 1g and Extended Data Fig. 1e). This suggests that MET-2-dependent H3K9me2 precedes deposition of the H3K9me3 mark in WT muscle and that it is sufficient to repress these loci in muscle. In general, the genes derepressed in the *met-2*- and *met-2 set-25-*mutant muscle were heterochromatic non-muscle genes with little or no expression in WT muscle (Fig. 1h and Extended Data Fig. 1e; 65% reads per kilobase of transcript per million mapped reads (RPKM) < 5). The pattern was distinct from that in mutant embryos, for in *met-2* muscle many, but not all, derepressed genes were germline-specific (for example, the P-granule component *pgl-1*; Fig. 1h).

To determine whether the changes in gene expression in H3K9me-deficient animals had overt effects on tissue development, we investigated muscle integrity. In body-wall muscle, the functional equivalent of mammalian skeletal muscle^22^, we found defects in sarcomere morphology in both the *met-2* and *met-2 set-25* mutants but not in the animals with the *set-25* mutation (Extended Data Fig. 1f). The tissue defects manifested as uneven and broken actin–myosin filament structures and defective muscle function (Extended Data Fig. 1f,g). Thus, although muscles form in the absence of H3K9me despite the misexpression of tissue-specific genes, the muscle integrity is compromised.

## H3K9me undergoes dynamic changes during differentiation

**Fig. 2:**
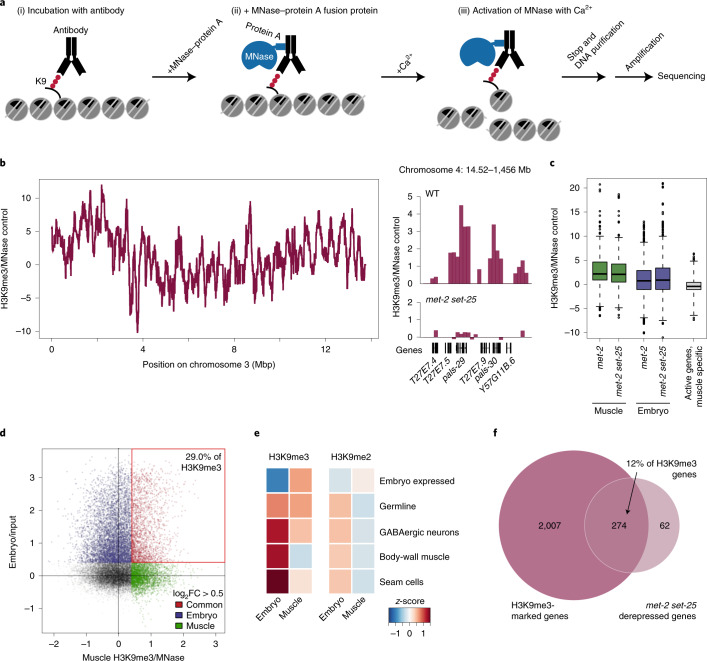
Changes in H3K9me during differentiation correlate with changes in gene expression. **a**, Schematic of the ChIC-seq technique. To control for unspecific MNase digestion, negative control samples were treated with MNase–protein A in parallel, but without antibody. **b**, ChIC-seq was performed on sorted muscle cells from WT L3 animals grown at 20 °C. Enrichment of H3K9me3 over the MNase control is shown along the entire length of chromosome 3 for WT worms (left). Genome browser view of H3K9me3 at the *pals-29* and *pals-30* locus on chromosome 4 in the WT and *met-2 set-25* mutants (right). **c**, Enrichment of H3K9me3 over the MNase control in muscle cells at genes derepressed in *met-2*- and *met-2 set-25*-mutant muscle cells and embryos compared with genes that are primarily expressed in muscle^20^ and all genes. Boxplots show the median (horizontal line), 25th to 75th percentiles (boxes), and 90% (whiskers) of the group. **d**, Correlation between the mean log_2_-transformed H3K9me3 levels at genes in embryos (ChIP–seq H3K9me3/input) versus L3 muscle cells (ChIC-seq H3K9me3/MNase control). Individual genes are coloured based on H3K9me3 enrichment in either tissue or in both. Muscle and embryos have 29% H3K9me3-marked loci in common (genes). **e**, Heatmap showing the mean levels of H3K9me3 (left) and H3K9me2 (right) in embryos and muscle cells at genes expressed in embryos, specifically expressed in the germline, GABAergic neurons, body-wall muscles and seam cells. The tissue-specific gene sets are from Cao et al.^20^, with the exception of the embryo expressed set, which is defined as RPKM > 8 in WT early embryos. **f**, Venn diagram showing the overlap between H3K9me3 over the MNase control in WT muscle cells, mapped by ChIC-seq, to the genes derepressed in *met-2 set-25*-mutant muscle cells normalized to the WT (Fig. 1e; FDR < 0.01, FC > 2; *n* = 2 independent biological replicates).

**Extended Data Fig. 2:**
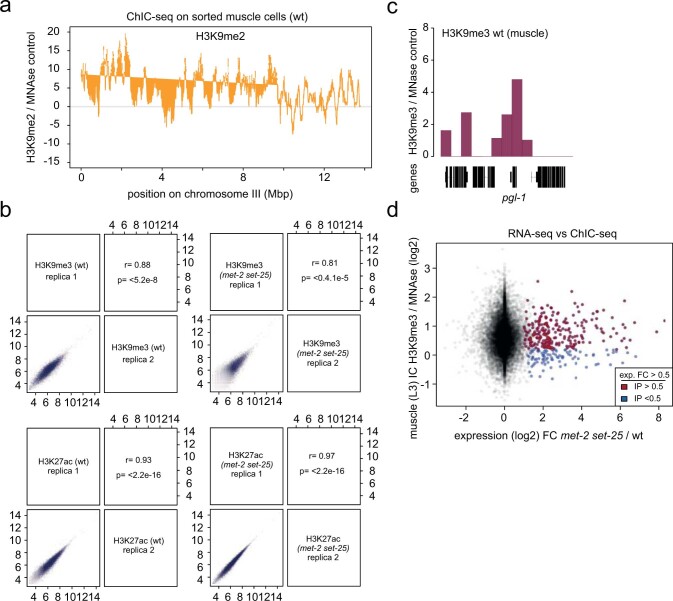
Mapping H3K9me2/me3 in muscle. a) ChIC-seq performed on sorted muscle cells from wt L3 animals grown at 20^o^C. Enrichment of H3K9me2 over an MNase control is shown along the entire chr III for wt worms. b) Correlations of ChIC-seq replica for H3K9me3 and H3K27ac in wt and *met-2 set-25* muscle cells. The corresponding R values represent spearman correlation coefficients. P-values are calculated using two-sided T-test. c) Genome browser view of H3K9me3 at the *pgl-1* locus in wt. d) Scatter plot correlating H3K9me3 over MNase control in muscle cells (ChIC-seq) compared with the mean log2FC of gene expression in *met-2 set-25* muscle cells over wt. Derepressed genes (Fig. 1e; FDR < 0.01, FC > 2) are coloured according to their enrichment for H3K9me3 in wt (red > 0.5, blue <0.5).

To confirm a direct role for H3K9me in tissue-specific gene repression, we checked whether the genes derepressed in the muscle of HMT mutants carried H3K9me3 in WT muscle. To this end, we mapped H3K9me2/me3 in isolated muscle cells using a modified CUT&RUN protocol (chromatin immuno-directed cleavage with sequencing, ChIC-seq)^23–25^ that was adapted for low cell numbers (Fig. 2a,b and Extended Data Fig. 2a,b). As expected, genes derepressed in *met-2-* or *met-2 set-25-*mutant muscle cells were enriched for H3K9me3 in WT muscle (Fig. 2b,c; for *pgl-1*, see Extended Data Fig. 2c). The genes derepressed in H3K9me-deficient embryos were less enriched for H3K9me3 in WT muscle, suggesting that H3K9me3 might not only be gained, but also be lost during development (Fig. 2c). The H3K9me3 ChIC-seq pattern in muscle was indeed substantially different from that of embryos, and only 29% of genes were found methylated at both stages (see results for chromatin immunoprecipitation with sequencing, ChIP–seq; Fig. 2d). The differences corresponded nicely to the distinct transcriptional programmes: genes expressed in WT embryos tended to gain H3K9me2/me3 in muscle (Fig. 2e), whereas genes that gained expression in muscle lost H3K9me2/me3. Genes expressed in other tissues, including germline cells, were tri-methylated at both stages of WT animals (Fig. 2e). Despite specific losses and gains of H3K9me during differentiation, only 12% of all H3K9me3-marked loci were derepressed following the loss of H3K9me in muscle (Fig. 2f; compare Fig. 1c and Extended Data Fig. 2d).

## H3K9me2 requires active maintenance in post-mitotic cells

**Fig. 3:**
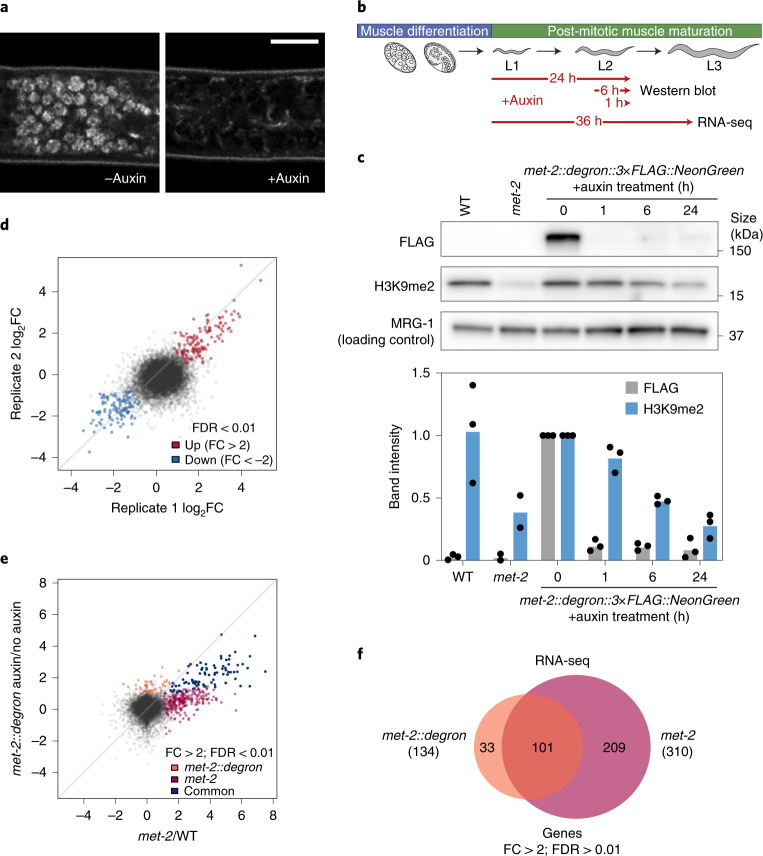
H3K9me2 requires active maintenance in post-mitotic muscle cells. **a**, Representative confocal images of MET-2 in the midsection of an L1 larva expressing MET-2::degron::3×FLAG::NeonGreen as well as a ubiquitously expressed TIR1 auxin receptor, after being seeded for 1.5 h on plates with (+) or without (−) auxin. Scale bar, 10 µm. Images represent two biologically independent experiments. **b**, Schematic of auxin addition for the MET-2::degron degradation experiments. For western blots, a synchronized population of worms was split onto different plates, with auxin added at different times during development. All samples were collected and processed simultaneously. For RNA-seq, a synchronized population was grown for 36 h in the presence or absence of auxin to obtain L3 larvae for muscle isolation. **c**, Example western blot of whole-cell lysates from WT as well as *met-2*-mutant and *met-2::degron* L2 larvae after growth at 20 °C with or without auxin for the indicated times (top). The membranes were blotted for FLAG, H3K9me2 and MRG-1 (as a control). The protein levels were normalized to endogenous MRG-1 and the control (0 h auxin) for *n* = 3 biologically independent experiments. **d**, Gene expression was determined for auxin treated over untreated sorted muscle cells isolated from L3 larvae expressing *met-2::degron*, after growth for 36 h at 20 °C with or without auxin. Loci identified as significantly changed are highlighted in colour (FDR < 0.01 and FC > 2, or < −2); *n* = 2 independent biological replicates. **e**, Correlation between gene expression (log_2_FC) in sorted *met-2::degron* muscle cells with auxin over no auxin and *met-2* (*met-2* null allele) over WT. Loci that were significantly changed (FDR < 0.01 and FC > 2) are coloured according to the genotype and significantly changed loci in both are coloured in dark blue. **f**, Venn diagram showing numbers of significantly derepressed genes from sorted muscle cells of *met-2::degron* animals (auxin treatment versus no auxin) and *met-2* (*met-2* null versus WT) and their overlap. Source data

**Extended Data Fig. 3:**
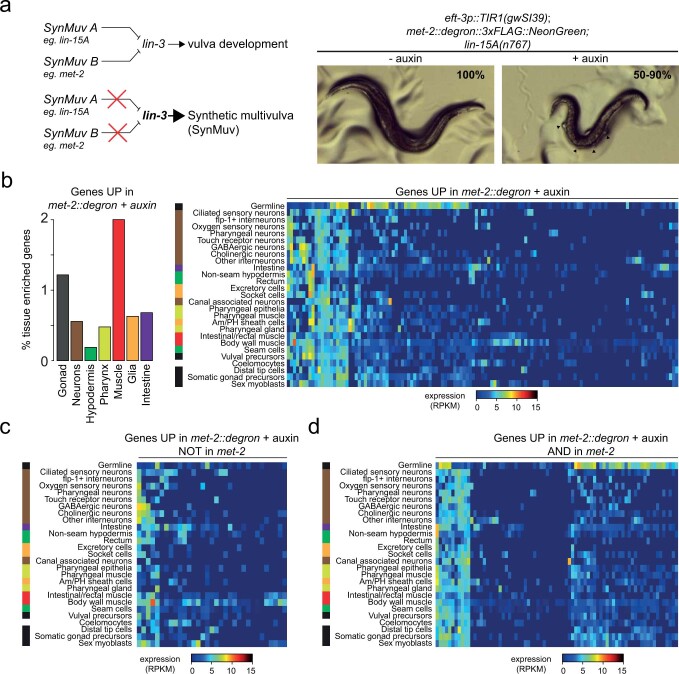
Acute depletion of MET-2 has similar effects as constitutive mutant. a) *Left*, scheme depicting classical synthetic multi-vulva (SynMuv) assay. Two independent pathways, involving SynMuv A and SynMuv B genes, independently repress *lin-3* expression to restrict vulval development. Only in the absence of both silencing mechanisms do worms develop the SynMuv phenotype. *Right*, Representative images of worms expressing MET-2::degron::3xFLAG::NeonGreen and a ubiquitous TIR1, crossed with *lin-15A(n767)*. Gravid adults were briefly transferred to plates with (+) or without (-) auxin, and their progeny were scored for SynMuv phenotype. Arrowheads indicate multiple vulvae on an auxin treated worm. All worms grown without auxin had a wild-type phenotype, while 50-95% of worms grown in the presence of auxin were SynMuv (N = 3 biological replicates, n pooled worms analysed; control=284, auxin=648). b) Genes derepressed in *met-2*::degron with auxin are plotted as a percentage of all tissue-enriched genes, (absolute number of genes: Gonad=49, Neurons=17, Hypoderm=3, Pharynx=7, Muscle=20, Glia=10, Intestine=16), *left*, or as a heatmap of individual derepressed gene (columns), in each cell type (row), *right*. c) Heatmap of individual derepressed genes (columns), in each cell type (row) for genes derepressed exclusively in muscle from *met-2::degron* with auxin and not in the *met-2* mutant. d) Same as (c), but for common genes.

To determine whether the active deposition of H3K9me continues in non-dividing differentiated cells, we investigated whether there is a requirement for MET-2 protein in post-embryonic tissues. The fusion of a degron tag to the endogenous MET-2 resulted in its rapid degradation in all larval tissues following the addition of auxin (<1 h; Fig. 3a–c). Degradation of MET-2 was accompanied by a progressive loss of H3K9me2, reaching the level of the constitutive *met-2* null allele by 24 h post auxin addition (Fig. 3b,c). As the majority of larval cells are post-mitotic^26^, H3K9me2 loss cannot be due to histone replacement during replication. As proof that MET-2 degradation impacts the organismal phenotype, we found that auxin-induced degradation of MET-2 in larvae yields a multi-vulva phenotype in combination with a *lin-15A* mutant, phenocopying the *met-2* deletion mutant (SynMuv assay; Extended Data Fig. 3a)^27^. We conclude that H3K9me requires constant maintenance by MET-2 HMT activity to maintain differentiated tissue identity.

To determine whether the post-embryonic loss of H3K9me2 affected the same genes as the constitutive *met-2* null, we treated synchronized early L1 larvae with auxin and isolated messenger RNA from L3 muscle cells. In L1-stage larvae, 81 of the 95 body-wall muscle cells are post-mitotic and seven undergo one final cell division^26^. Of the 134 genes derepressed with auxin treatment, 76% overlapped with the genes derepressed in the *met-2*-mutant muscle (Fig. 3d–f and Extended Data Fig. 3b–d). The auxin-sensitive genes also contain germline-expressed genes (Extended Data Fig. 3c,d); thus, their derepression cannot reflect a failure in germline-to-soma transition as previously proposed^28,29^. We conclude that terminally differentiated post-mitotic cells actively reinforce H3K9me2 to repress genes of other cell lineages, including the germline.

## Gene derepression correlates with specific TF motifs

**Fig. 4:**
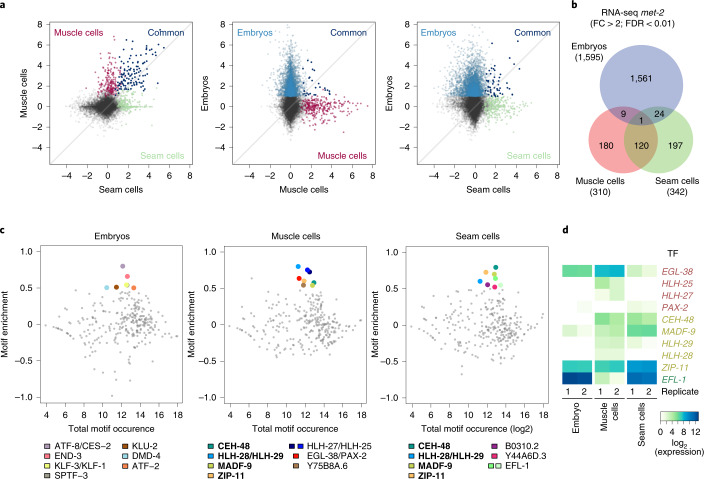
A tissue-specific TF repertoire correlates with gene derepression in the absence of H3K9me. **a**, Correlation between the gene expression (log_2_FC) of *met-2* over WT from sorted muscle cells, sorted seam cells, or early embryos, as marked. Loci that were significantly changed compared with the WT (FDR < 0.01 and FC > 2) are coloured-coded by tissue, and loci that were significantly changed in both tissues in the comparison are coloured in dark blue. **b**, Overlap of significantly derepressed genes from sorted muscle cells, sorted seam cells or early embryos of *met-2* mutants versus WT (as in **a**). **c**, Enrichment (log_2_-transformed) of TF motifs in the promoters of the indicated derepressed gene sets (**a**) versus log_2_-transformed genome-wide occurrence. Significantly enriched motifs (log_2_ enrichment > 0.5 and log_2_ abundance > 8) are colour-coded with their corresponding TF(s) indicated. Motifs that are enriched in both tissues are shown in bold font. **d**, Expression levels of TFs that have enriched motifs in the promoters of genes derepressed in muscle (red), seam cells (green) or both (gold). TF expression from RNA-seq, with two biologically independent replicas per tissue shown.

**Extended Data Fig. 4:**
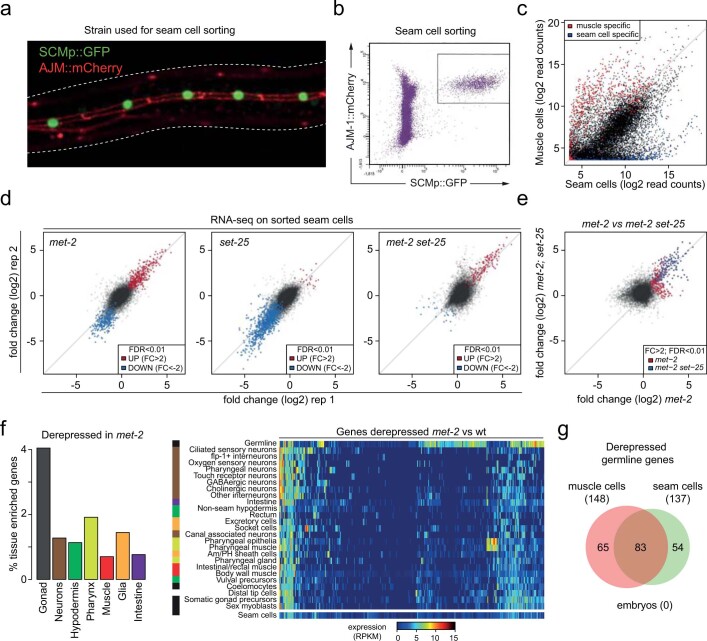
Derepression of genes in seam cells deficient for met-2. a) Representative image of an L3 worm with GFP expressed from the seam cell specific SCM promoter and cell junction marker AJM-1::mCherry. b) Representative FACS plot showing gating strategy for GFP, mCherry double positive cells (seam cells), following a similar gating strategy as muscle cells (Extended Data Fig. 1c), including exclusion of DRAQ7 + dead cells. c) Scatter plot showing gene expression as log2 of read counts from sorted muscle cells (y-axis) or seam cells (x axis) isolated from wt L3 worms grown at 20^o^C. Individual muscle specific (red) or seam cell specific (blue) genes (based on scRNAseq in Cao et al., 2017) are highlighted. N = 3. d) Scatter plot showing gene expression as log2FC of indicated mutants over wt, from sorted seam cells. Loci identified as significantly changed are highlighted in colour (FDR < 0.01 and FC > 2, red or < -2, blue). Two independent biological replicas shown. e) Correlation between the log2FC over wt for gene expression between *met-2* or *met-2 set-25*. Loci significantly changed compared to wt (FDR < 0.01 and FC > 2 or < -2) are coloured according to the genotype and loci significantly changed in both mutant genotypes are coloured in dark purple. f) Genes derepressed in *met-2* seam cells are plotted as a percentage of all tissue-enriched genes, *left*, or as a heatmap of individual derepressed gene (columns), in each cell type (row), *right*. (absolute number of genes: Gonad=160, Neurons=49, Hypoderm=18, Pharynx=26, Muscle=16, Glia=22, Intestine=27). g) Venn diagram comparing overlap of significantly derepressed germline genes from sorted muscle cells and sorted seam cells of *met-2* mutants over wt. Note: there were no significantly derepressed germline genes in embryos.

To determine whether the set of genes that becomes derepressed by loss of MET-2 in differentiated larvae is cell-type specific, we examined the transcriptome of hypodermis-derived seam cells from L3 larvae of WT and H3K9 HMT mutants (Extended Data Fig. 4a–c). In FACS-sorted seam cells^30^, as in muscle cells, gene silencing was almost entirely dependent on MET-2, and the genes that were derepressed were characteristic of germline or other tissues (Extended Data Fig. 4d–f). Interestingly, only 39% of the genes derepressed in H3K9me-deficient muscle were affected in seam cells (35% vice versa; Fig. 4a,b) and although germline genes were affected in both tissues, these were partially distinct sets of germline-specific genes (Extended Data Fig. 4g). Furthermore, there was little overlap between the genes derepressed in embryos and those derepressed in either tissue (Fig. 4a,b). We conclude that the function of H3K9me-marked heterochromatin in gene silencing is both cell-type and developmental-stage specific.

**Extended Data Fig. 5:**
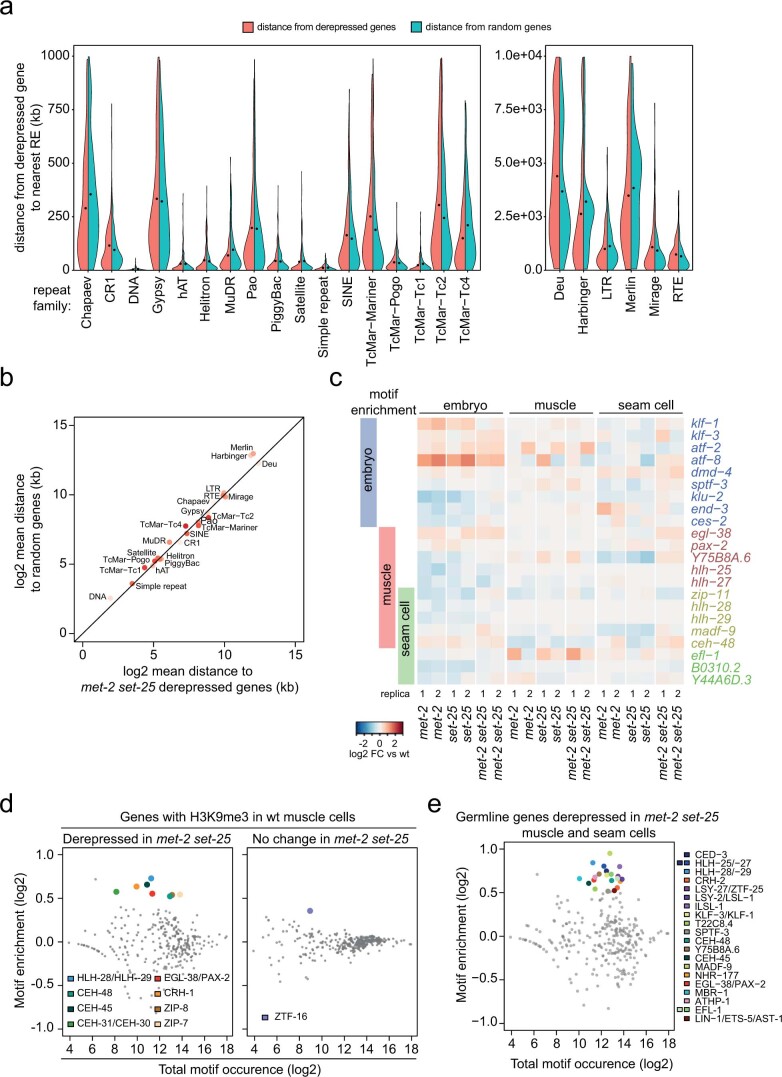
Proximity to repeats does not correlate with gene derepression in H3K9me-deficient muscle cells. a) Violin plots of distances (in kb) between derepressed genes and the nearest repetitive element subdivided by repeat family (according to repeat masker annotation), in muscle cells from *met-2 set-25* mutants (red). Derepressed gene sets are compared to the same number of random, non-derepressed genes (cyan). Plots have been split to accommodate range of distances. The mean distances for each group are indicated by a dot. Note that repeat elements Chapeav, SNA, TcMar-Tc4, Harbinger and Merlin are slightly closer to the TSS of derepressed genes than the random gene set, but for all except DNA, the average distance to the TSS is >200 kb. b) Correlation of the mean distances in log2 kb between genes derepressed in met-2 set-25 mutants and random set of genes to the nearest repetitive element subdivided by repeat family. We note that the smallest mean distance (DNA repeat family) was still 3.9 kb upstream of the TSSs. Only 87 (26%) of the 331 derepressed genes have a repeat element < 1 kb from the corresponding TSS. In comparison 34% of all genes in the genome have a repeat element < 1 kb in proximity to their TSS. c) Heatmap highlighting log2FC of gene expression for the indicated TFs, in two independent replicas, with indicated mutants over wt. d) Plot showing log2 enrichment of TF binding motifs, versus genome-wide occurrence, in genes marked by H3K9me3 in wt and transcriptionally derepressed (FC > 2; FDR < 0.01) in *met-2 set-25* muscle cells (labelled “derepressed”) or transcriptionally unchanged (labelled “no change”). Significantly enriched motifs (log2 enrichment>0.5; log2 abundance>8) are colour coded with their corresponding TF(s) indicated. e) As (d) for germline genes that are derepressed in both muscle and seam cells from *met-2 set-25* animals.

To gain mechanistic insight into what drives gene derepression following the loss of H3K9me, we asked what characterizes the derepressed sets of genes. Although aberrantly expressed REs can affect the expression of proximal genes^31^, we found no preferential proximity of the genes derepressed in *met-2 set-25*-mutant muscle cells to REs (Extended Data Fig. 5a,b). We examined the enrichment of TF binding motifs^32^ in the promoters of derepressed genes from embryos, muscle and seam cells versus all promoters genome-wide. The comparative occurrence of motifs related to 226 *C. elegans* TFs revealed a unique set of TF motifs enriched in the promoters derepressed in embryos as compared to muscle or seam cells, whereas the enriched TF motifs in muscle and seam cells showed partial overlap (Fig. 4c). Importantly, the expression of the relevant TFs themselves was not altered by HMT mutation (except for *atf-8* in embryos; Extended Data Fig. 5c). Interestingly, most of the TFs that bind motifs upstream of derepressed genes in muscle or seam cells are not expressed in embryos, with the exception of EGL-38, ZIP-11 and EFL-1. We found four other TFs to be expressed in both muscle and seam cells, four to be predominantly muscle specific and two that were expressed most strongly in seam cells (Fig. 4d).

The embryo-specific TF motifs enriched on *met-2-*sensitive genes were recognized by factors that have homologues active in early mammalian development, namely ATF-8/CES-2 (TEF)^33^ and KLF-1 (KLF6)^34^, whereas the muscle- and seam cell-specific TFs have homologues involved in tissue determination, for example, CEH-48 (Onecut1/2), which in mammals is a central regulator of both differentiation and the maintenance of the differentiated state of liver and pancreas cells as well as retinal neurons^35,36^. Others involved in tissue determination are HLH-25, -27, -28 and -29 (all homologous to HES1)^37,38^ and EGL-38 and PAX-2 (PAX-2/PAX-5)^39^. When we restricted our analysis to the derepressed genes in mutant muscle that were H3K9me3-marked in the WT, we found that the same TF motifs were enriched. In contrast, no TF motifs were enriched for H3K9me3-marked non-derepressed genes (Extended Data Fig. 5d). The fact that the enriched TF motifs are largely shared between muscle and seam cells, even though two-thirds of the expressed genes are different, suggests that these TFs may act together with co-factors that determine tissue-specific gene expression following the loss of H3K9me.

## H3K9me restricts specific TF binding and activity

**Fig. 5:**
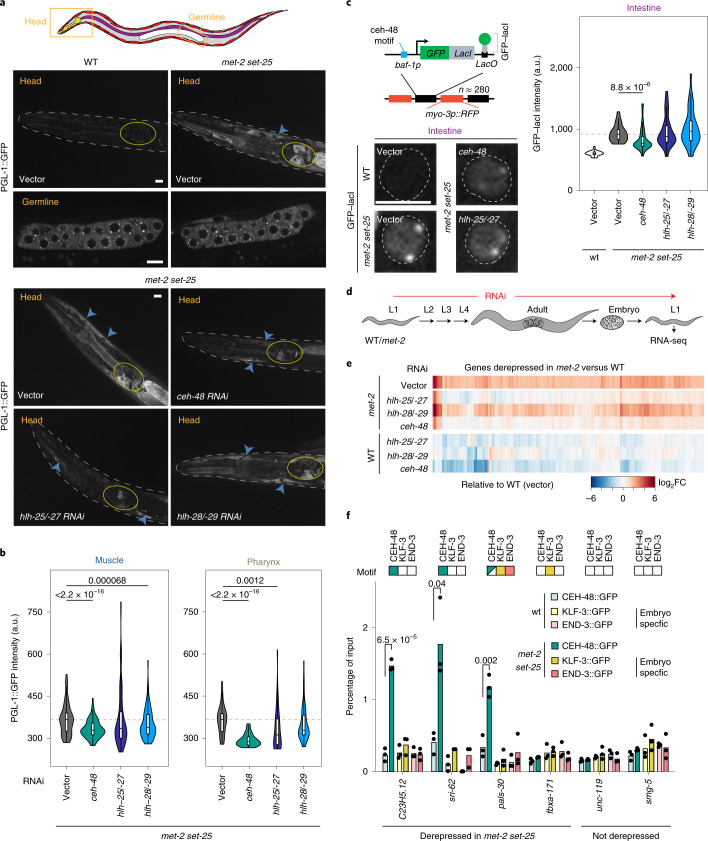
Binding of specific TFs is necessary for gene derepression by H3K9me loss. **a**, Schematic of an L3 worm highlighting the head and germline regions (top). Representative images of endogenously tagged PGL-1::GFP in these regions of WT or *met-2 set-25*-mutant L3 larvae after the indicated RNAi (vector, control). Muscle (blue arrowheads) and pharynx (yellow circles) cells are indicated. Scale bars, 10 µm. **b**, PGL-1::GFP intensity in the indicated tissues of *met-2 set-25*-mutant L3 larva. The grey dotted line indicates the median of the vector-treated *met-2 set-25* larvae. Muscle: *n* = 286 (vector), 649 (*ceh-48*), 372 (*hlh-25*/*-27*) and 538 (*hlh-28*/*-29*) cells; pharynx, *n* = 94 (vector), 390 (*ceh-48*), 168 (*hlh-25*/*-27*) and 269 (*hlh-28*/*-29*) cells; pooled from two biological independent experiments. PGL-1::GFP intensities that differed significantly from the vector control RNAi are indicated. **c**, Schematic of the *gwIs4* heterochromatic reporter with a CEH-48 motif in the promoter (top left). Representative images of GFP–LacI in intestinal nuclei of WT or *met-2 set-25, cec-4::WmCherry;gwIs4* L1 animals grown at 20 °C with the indicated RNAi (bottom left). The nuclear periphery is outlined. Scale bar, 5 µm. Mean GFP–LacI intensity per nucleus in intestinal cells (right). The grey dotted line indicates the median of the vector-treated *met-2 set-25* larvae. Data from one of two representative experiments. Statistics were derived from the number of cells analysed in each group: *n* = 31 (WT vector), 38 (*met-2 set-25* vector), 46 (*ceh-48*), 40 (*hlh-25*/*-27*) and 37 (*hlh-28/29*) cells. GFP::LacI intensities that differed significantly from the vector control RNAi are indicated. **b**,**c**, Two-sided Wilcoxon signed-rank test. Boxplots show the median (horizontal line), 25th to 75th percentiles (boxes), and 90% (whiskers) of the group; a.u., arbitrary units. **d**, Schematic for RNAi knockdown of TFs in WT or *met-2* animals. **e**, Heatmap showing the gene expression (log_2_FC) for the indicated RNA in either WT or *met-2*-mutant animals normalized to the WT treated with vector. Displayed genes are those that were derepressed in vector-treated *met-2* animals over the WT (FDR < 0.01, FC > 3); *n* = 3. **f**, ChIP–qPCR of GFP-tagged TFs in WT and *met-2 set-25* L3 larvae. Target genes were selected based on derepression in *met-2 set-25* L3 muscle and total L1 as well as absence of expression in WT larval tissues (Extended Data Fig. 6i and ref. ^21^). The presence of TF motifs in target genes is indicated. The *pals-30* promoter has two 8/9 nucleotide matches to the CEH-48 motif and seven 6/9 nucleotide matches, indicated by half shading; *n* = 3 biological independent experiments. *P* values derived from a two-sided *t*-test are indicated. Source data

It remained to be shown that the TFs enriched at derepressed genes were responsible for the observed gene derepression following the loss of H3K9me. To achieve this, we knocked down muscle-enriched TFs one by one and monitored the impact of their loss on *met-2* targets. One locus we monitored was *pgl-1*, chosen because it is expressed in mutant muscle and seam cells, carries H3K9me3 in WT larvae (Extended Data Fig. 2c) and has motifs for multiple relevant TFs in its promoter, including HLH-25/-27, HLH-28/-29, CEH-48 and MADF-9 (Supplementary Table 3), like many derepressed germline genes (Extended Data Fig. 5e). Using quantitative microscopy, we measured expression from the endogenous *pgl-1* locus by means of a PGL-1::green fluorescent protein (GFP) fusion^40^. In WT animals, PGL-1::GFP was expressed only in the germline, as expected^41,42^, whereas in animals with the *met-2 set-25* double mutation, PGL-1::GFP was expressed in multiple tissues, including muscle and pharynx (Fig. 5a).

**Extended Data Fig. 6:**
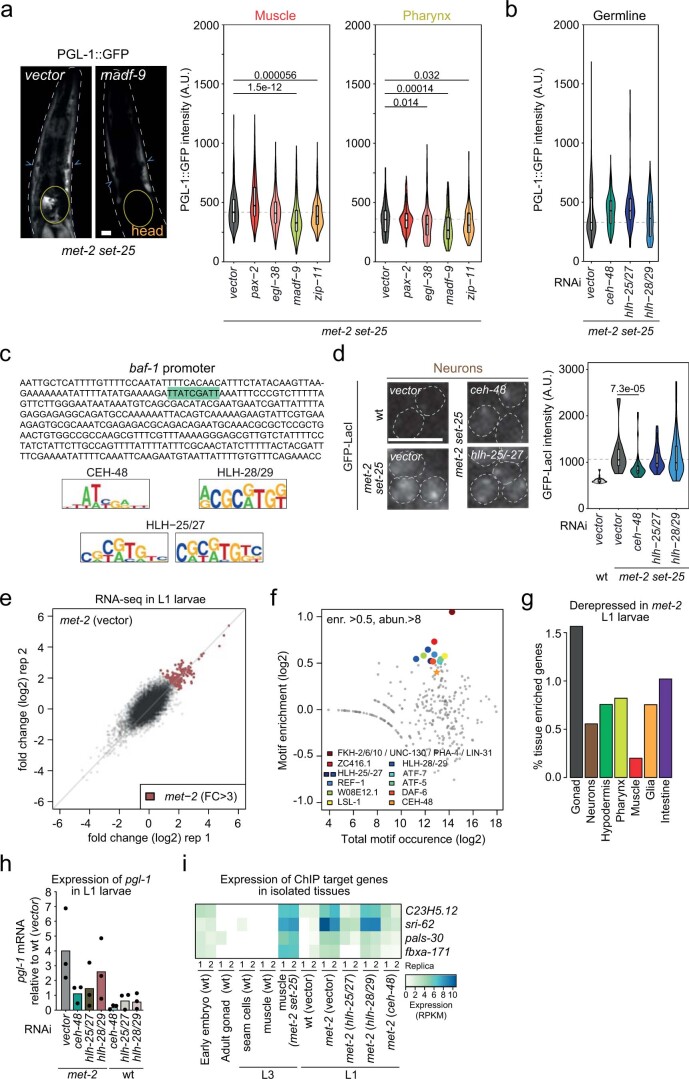
Derepression of *pgl-1* in the absence of H3K9me is dependent on specific TFs. a) *Left*, representative images of PGL-1::GFP in the head of *met-2 set-25 larvae* with indicated RNAi. Muscle (blue arrows) or pharynx (yellow circles) are indicated. Scale bars, 10 µm. *Right*, quantifications of PGL-1::GFP intensity from indicated tissues. Grey bar indicates median from *met-2 set-25(vector)*. n(pooled cells from 2 biological independent replicas)= Muscle (*vector*:440; *pax-2*:291; *egl-38*:363; *madf-9*:275; *zip-11*:357), Pharynx (*vector*:201; *pax-2*:171; *egl-38*:229; *madf-9*:121; *zip-11*:176). p-values of PGL-1::GFP intensities significantly down vs. vector RNAi represent two-sided Wilcoxon signed-rank test. b) Quantifications of PGL-1::GFP intensity in germline. Grey bar indicates median from vector-treated *met-2 set-25*. n(pooled cells from 2 biological independent replicas)*vector*:300; *ceh-48*:400; *hlh-25/-27*:235; *hlh-28/29*:345. PGL-1::GFP intensities are not significantly changed vs vector control (two-sided Wilcoxon signed-rank test). c) *Top*, Sequence of *baf-1* promoter upstream of *gfp::lacI* in *gwIs4* reporter. CEH-48 motif is highlighted. *Bottom*, sequence logos for CEH-48, HLH-25/-27 and HLH-28/-29. d) Representative images of GFP–LacI in neurons from wt or *met-2 set-25, cec-4::WmCherry; gwIs4* L1 animals with indicated RNAi. Nuclear periphery is highlighted. Scale bar, 5 µm. *Right*, Quantifications of mean GFP–LacI intensity per nucleus. Grey bar indicates median from *met-2 set-25(vector)*. Statistics were derived from n(cells)= wt(*vector)*:31; *met-2 set-25(vector*):38; (*ceh-48*):48; (*hlh-25/-27*):50; (*hlh-28/29*):46, collected from one out of two independent experiments with similar results. p-values from GFP::LacI intensities significantly different from vector RNAi represent two-sided Wilcoxon signed-rank test. a,b,d) Boxplots show median, boxes 50% and whiskers 90% of groups. e) Scatter-plot showing gene expression as log2FC for *met-2* over wt, from whole L1 worms on vector RNAi. Significantly increased loci (FDR < 0.01, FC > 3) are highlighted, red. f) Log2 enrichment of TF motifs in promoters of derepressed genes versus genome-wide occurrence. Enriched motifs (log2 enrichment>0.5, log2 abundance>8) are colour coded according to TF(s). Though not above this cut-off, CEH-48 (*) is included. g) Genes derepressed in *met-2*(*vector*) are plotted as a percentage of all tissue-enriched genes. (n(genes): Gonad=63, Neurons=21, Hypoderm=12, Pharynx=12, Muscle=2, Glia=18, Intestine=24). h) Expression (RPKM) *pgl-1* (RNA-seq) from wt and *met-2* mutant L1 larvae treated with RNAi against *ceh-48*, *hlh-25/-27*, *hlh-28/-29* and empty-vector. N = 3 biological independent replicas. i) Expression (RPKM) for ChIP target genes (rows) using RNA-seq data from isolated tissues and total L1 larvae treated with RNAi against *ceh-48*, *hlh-25/-27*, *hlh-28/-29* and empty-vector (RNAi indicated in brackets). Source data

In these H3K9me-deficient worms, RNA interference (RNAi) against *ceh-48* strongly reduced PGL-1::GFP derepression in both muscle and pharynx cells, while RNAi against *hlh-25*/*-27, hlh-28*/*-29, zip-11* and *madf-9* each had a partial effect (Fig. 5a,b and Extended Data Fig. 6a). Reflecting the absence of their motifs and/or TF expression, *pax-2* and *egl-38* RNAi had almost no effect on *pgl-1* expression in muscle or pharynx cells. We also detected no reduction in GFP signal in the germline, where *pgl-1* is not repressed by H3K9me (Fig. 5a and Extended Data Fig. 6b).

To further confirm that tissue-specific TFs are required for *met-2*-dependent gene derepression in larval tissues, we made use of a well-established heterochromatic reporter, *gwIs4* (ref. ^21^), which contains a *baf-1* promoter with a binding site for CEH-48 and none for HLH-25/-27 or HLH-28/-29 (Extended Data Fig. 6c). The *baf-1p* promoter drives expression of GFP–LacI in this integrated reporter array, which is normally heterochromatinized and repressed^13^. Earlier work showed widespread GFP–LacI expression following the loss of H3K9me^13,21^. Consistent with the results for *pgl-1*, *baf-1p::gfp–lacI* derepression in *met-2 set-25*-mutant animals was sensitive to RNAi against *ceh-48* (Fig. 5c and Extended Data Fig. 6d) but insensitive to RNAi for *hlh-25*/*-27* or *hlh-28*/*-29*.

To document the effects of TF ablation on a genome-wide scale, we performed RNA-seq on whole L1 WT and *met-2*-mutant larvae treated with RNAi against individual TFs of relevance (Fig. 5d). We chose L1-stage animals so that we could see the effect of RNAi without potentially confounding developmental defects. In *met-2* mutants grown on bacterial carrying an empty RNAi vector we observed derepression of 171 genes representing a broad range of tissues, with enrichment for many TF motifs found in muscle, including HLH-25/-27, HLH-28/-29 and CEH-48 more weakly (Extended Data Fig. 6e–g). Confirming a role for HLH-25/-27 and CEH-48 in H3K9me-target derepression, RNAi against these factors broadly suppressed the upregulation triggered by the loss of H3K9me, whereas HLH-28/-29 had a more selective effect (Fig. 5e). Once again, the levels of *pgl-1* mRNA were elevated in the *met-2* mutant, and this was sensitive to TF knockdown (Extended Data Fig. 6h). This confirms that specific TFs are required to induce the aberrant gene expression that occurred following the loss of H3K9me in differentiated tissues.

The final step was to examine whether H3K9me blocks the binding of such TFs to their cognate promoters. We performed ChIP in WT and *met-2 set-25*-mutant L3 larvae expressing GFP-tagged CEH-48 and used similar GFP fusions to the embryo-specific TFs KLF-3 and END-3 as negative controls. Promoter binding was measured by ChIP coupled to detection by quantitative PCR (qPCR) at a subset of target genes that were derepressed in *met-2 set-25*-mutant muscle cells and are expressed in WT embryos^20^ but not in WT larval or adult tissues (Extended Data Fig. 6i, see legend). We have indicated above the graph in Fig. 5f whether binding motifs for the relevant TFs are present in the promoter assayed. We found that CEH-48 gained binding to the promoters of the three derepressed genes bearing CEH-48 motifs in the *met-2 set-25* mutants but not to a promoter lacking its motif or to control genes that were not upregulated (Fig. 5f). No binding was detected in the WT animals, presumably because H3K9me blocked the promoter. As expected, the negative controls KLF-3 and END-3 did not bind in either the WT or *met-2 set-25* mutants (Fig. 5f). These data argue that H3K9me prevents the unscheduled binding of at least one of the TFs found to drive gene derepression following the loss of H3K9me in differentiated tissues.

## Gene derepression does not require decompaction

**Fig. 6:**
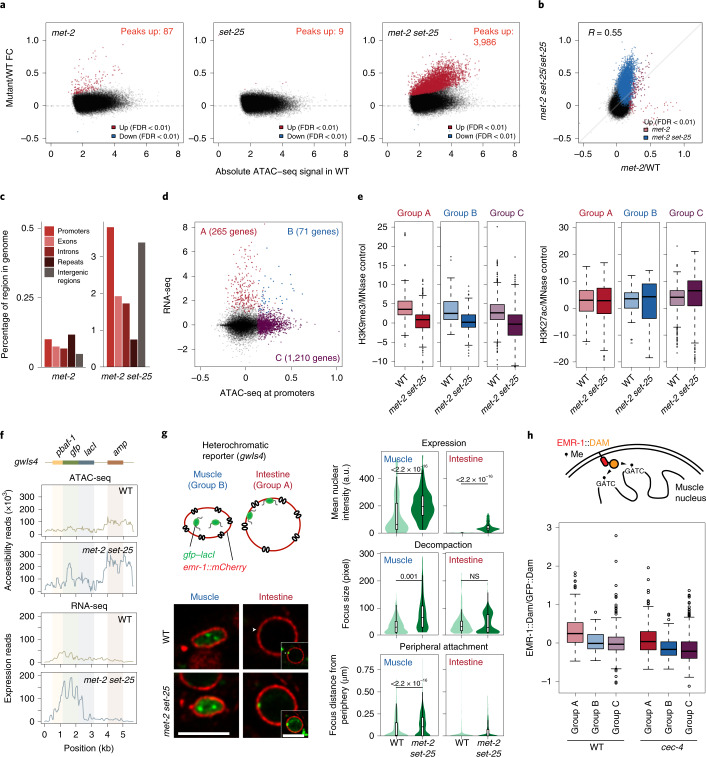
Enhanced accessibility in the absence of H3K9me is not necessary for gene expression. **a**, MA plots for ATAC-seq peaks in sorted L3 muscle cells, with the mean log_2_FC of the indicated HMT mutants over the WT versus the absolute signal in WT cells (log2 counts per million (CPM)). Peaks that are significantly more (up; FDR < 0.01) and less (down; FDR < 0.01) accessible are highlighted. Dashed grey line represents no change. The number of peaks that gain accessibility are indicated. The mean log_2_FC was calculated from *n* = 3 independent biological replicates. **b**, Correlation between the log_2_FC for accessibility between *met-2*/WT and *met-2 set-25*/*set-25*. Significantly changed loci (FDR < 0.01) are coloured based on genotype and common loci in maroon. **c**, Percentage (relative to genome-wide occurrence) of promoters, exons, introns, repeats and intergenic regions with significantly increased accessibility in *met-2*- (left) and *met-2 set-25*-mutant (right) muscle cells. **d**, Correlation between the mean gene expression (log_2_FC) and promoter accessibility (log_2_FC) in muscle cells from *met-2 set-25* mutants normalized to the WT. Individual genes are highlighted and divided into groups based on increased expression (Group A), increased accessibility (Group C) or both (Group B). **e**, H3K9me3 (left) and H3K27ac (right) ChIC-seq enrichment (over the MNAse control) at Group A, B and C genes in WT and *met-2 set-25*-mutant muscle cells. **f**, GFP–LacI cassette from the *gwIs4* heterochromatic reporter (top). The plots show the normalized average counts of ATAC-seq (middle) and RNA-seq (bottom) reads across the GFP–LacI cassette in WT and *met-2 set-25*-mutant muscle cells. **g**, Schematic of muscle and intestine nuclei with GFP–LacI foci from the *gwIs4* reporter and *emr-1::mCherry* marking the nuclear periphery (top left). Images of muscle and intestine cells from WT and *met-2 set-25*-mutant L1 animals (bottom left). Due to the low level of GFP–LacI expression in the intestine, insets with increased brightness are included. Scale bars, 5 µm. GFP–LacI nuclear intensity, focus size and focal distance from the nuclear periphery (right). Muscle, *n* = 332 (WT) and 651 (*met-2 set-25*); and intestine, *n* = 346 (WT) and 454 (*met-2 set-25*) pooled foci from two biological independent experiments. NS, not significant (*P* > 0.05); two-sided Wilcoxon signed-rank test. a.u., arbitrary units. **h**, Schematic of Emerin DamID, wherein EMR-1 is fused to DAM methylase^49^ (top). EMR-1::Dam methylation enriched over GFP::Dam (log_2_-transformed) control in WT and *cec-4*-mutant muscle cells at Group A, B and C genes (bottom). Data points represent 10 kb bins; *n* = 3 biological independent replicas. Note that CEC-4 protein tethers H3K9me-modified heterochromatin at the nuclear periphery^49,59^. **e**,**g**,**h**, Boxplots show the median (horizontal line), 25th to 75th percentiles (boxes), and 90% (whiskers) of the group.

H3K9me is associated with condensed chromatin; thus, the reduced accessibility of heterochromatic promoters has been proposed to restrict TF binding^43–46^. To investigate whether we could detect increased accessibility following the loss of H3K9me, we performed a tissue-specific ATAC-seq assay. Using WT and HMT-deficient muscle cells, we confirmed that H3K9me compacts chromatin: we monitored increased Tn5 accessibility at 3,986 peaks in the *met-2 set-25* double mutant (Fig. 6a; FDR < 0.01). The effects were far less pronounced in the single mutants (*met-2*, 87 peaks; and *set-25*, 9 peaks; Fig. 6a). Although the accessibility changes in the *met-2*- and *met-2 set-25*-mutant strains showed the expected positive correlation (Fig. 6b; *R* = 0.55), there was a striking discrepancy between the changes in gene expression and ATAC-seq. Genes derepressed in muscle were almost identical between the *met-2* and *met-2 set-25* mutants (Fig. 1g), whereas the ATAC-seq signal increase was far more pronounced in the double mutant. This suggested that SET-25 alone (that is, in the *met-2* single mutants) is sufficient to restrict ATAC-seq sensitivity, albeit not to repress transcription.

**Extended Data Fig. 7:**
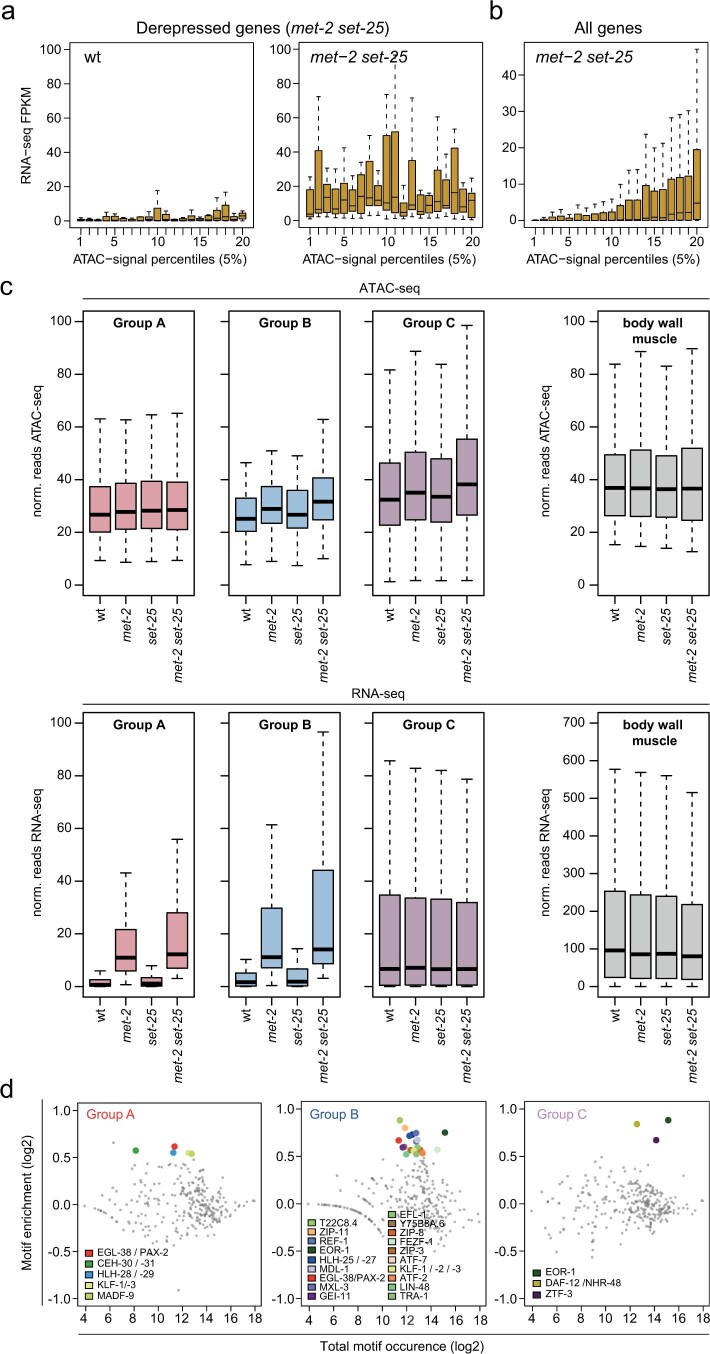
Characteristics of Group A, B and C genes. a) Interquartile box plot comparing RNA expression levels with increasing promoter accessibility in muscle cells, based on wt ATAC-seq signal, binned as 5 percentile ranges. Comparisons are done using the gene set derepressed in *met-2 set-25* muscle cells and using RNA-seq from wt or *met-2 set-25*. b) Same as (a), comparing *met-2 set-25* expression and wt accessibility, at all genes. c) The normalized ATAC-seq reads (*top)* or expression as RPKM from RNA-seq (*bottom*) from muscle cells of indicated genotypes are plotted, as boxplots, for the gene sets of Group A/B/C (Fig. 6d) or muscle specific genes^1^. a-c) Boxplots show median, boxes 50% and whiskers 90% of the group. d) Plots showing log2 enrichment of TF motifs in promoters of gene sets from different groups (Fig. 6d) versus genome-wide occurrence (log2). Significantly enriched motifs (log2 enrichment>0.5, log2 abundance>8) are colour coded with their corresponding TF(s) indicated.

The regions that did gain accessibility in the *met-2* mutant were equally enriched for REs and promoters, whereas changes in the *met-2 set-25* double mutant mapped overwhelmingly to promoters and intergenic regions (Fig. 6c). However, even in the *met-2 set-25* mutants, the changes in accessibility did not correlate strictly with changes in gene expression (Extended Data Fig. 7a). This lack of correlation was specific to genes derepressed in the *met-2 set-25*-mutant muscle, for overall we scored the expected correlation between increased ATAC-seq signals and highly transcribed genes across the entire transcriptome (Extended Data Fig. 7b).

We next plotted the changes in RNA-seq (increased expression levels) versus changes in ATAC-seq (accessibility) triggered by the loss of H3K9me. The genes that were affected in muscle fell into three classes: the majority of derepressed genes (265/336 genes) did not gain ATAC-seq sensitivity at their promoters (Group A), whereas 22% (71/336) gained accessibility coincident with an increase in transcription (Group B; Fig. 6d). Surprisingly, 1,210 loci showed increased accessibility without increased transcription (Group C; Fig. 6d).

Surprisingly, the genes of both Group A and B had on average similar and low absolute levels of accessibility and expression in WT muscle (Extended Data Fig. 7c), whereas the Group C genes started with higher absolute levels of expression and accessibility in the WT muscle, albeit not as high as that scored for the muscle-expressed genes (Extended Data Fig. 7c). In addition, all three groups had comparable levels of H3K9me3 in the WT muscle, which was sensitive in all cases to the loss of *met-2* and *set-25* (Fig. 6e). However, the Group A genes did not acquire H3K27ac, a mark associated with active loci and enhancers^48^, in the *met-2 set-25* mutant, although it increased in Group C. The Group B genes had a weaker or more variable increase in H3K27ac (Fig. 6e).

In addition to endogenous loci, we also tracked the expression and accessibility of the *gwIs4* heterochromatic array in muscle (both increased in the *met-2 set-25* mutant; Fig. 6f). We could further quantify *gwIs4* compaction and subnuclear position by microscopy given that the integrated array contains *lacO* sites and can bind GFP::LacI (Figs. 5c and 6g)^13^. A comparison of WT and *met-2 set-25*-mutant muscle cells confirmed that *gwIs4* physical chromatin compaction and expression increase coordinately following the loss of H3K9me (Fig. 6g). In contrast, intestinal cells showed derepression without decompaction, resembling the Group A genes (Fig. 6g). This difference seemed to correlate with subnuclear position: measurements of the distances of the GFP::LacI spots to the nuclear periphery confirmed an internal position^21,49,50^ for the *gwIs4* array in muscle that shifted even more internally in the *met-2 set-25* mutant, whereas in intestine the reporter was strongly peripheral and stayed largely peripheral despite derepression (Fig. 6g). Although the repetitive nature of the reporter may be a confounding factor, the behaviour of *gwIs4* suggested that genes located in the nuclear interior might be more susceptible to chromatin decompaction (and Tn5 attack) following the loss of H3K9me, whereas genes at the nuclear periphery appear to be derepressed without measureable decompaction.

To determine whether this correlation applies to endogenous single-copy genes, we compared the association of Group A, B and C genes with the nuclear periphery using muscle-specific Emerin DNA adenine methyltransferase identification (DamID; Fig. 6h)^49^. The loci that gained accessibility (Groups B and C) indeed showed a more internal localization overall in WT muscle cells than genes that remained compacted during derepression (Group A). All positioning was sensitive to loss of the H3K9me anchor CEC-4. Together, we conclude that whereas ATAC sensitivity increases dramatically following the loss of H3K9me, the increase does not always correlate with expression. When it does, the genes are generally located away from the nuclear envelope.

## H3K9me restricts enhancer activation

Given the distinct behaviours of the loci in Groups A, B and C, we queried whether the promoters were enriched for distinct TF motifs. As expected, the Group A and B gene promoters were enriched for the TF motifs identified among the derepressed genes in muscle (Fig. 4c and Extended Data Fig. 7d), and the Group B genes also bound basic leucine zipper (bZIP) and zinc-finger TFs (Extended Data Fig. 7d). In the non-derepressed Group C, which nonetheless gained ATAC-seq sensitivity, the only motifs significantly enriched were for EOR-1, DAF-12/NHR-48 and ZTF-3. Importantly, all three of these factors have been shown to bind enhancers during *C. elegans* development^47,51^.

**Fig. 7:**
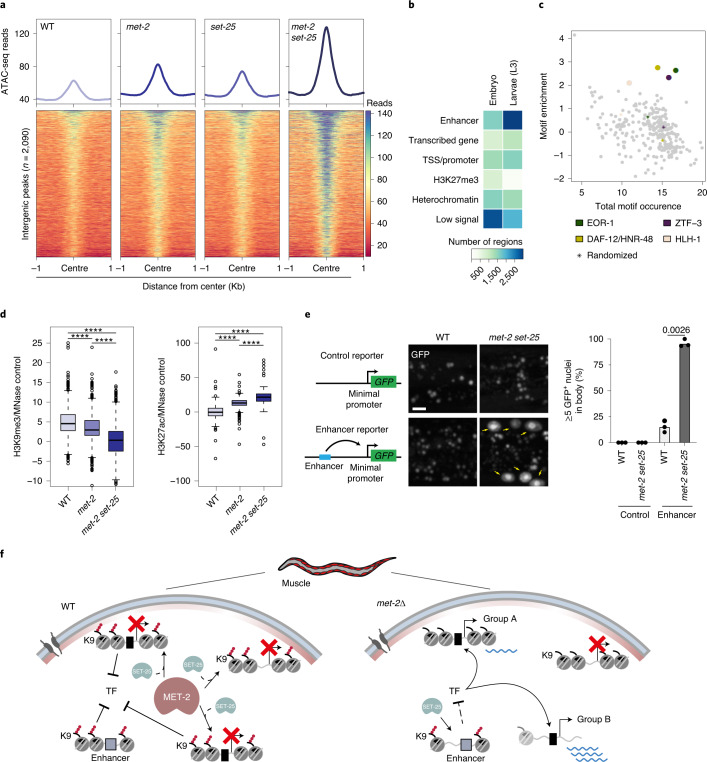
H3K9me can restrict enhancer accessibility and activity. **a**, Metaplots comparing the mean ATAC-seq reads at all intergenic peaks that were significantly more accessible in *met-2 set-25*-mutant muscle versus WT, anchored at the peak centre (top). Heatmap showing ATAC-seq reads over equally sized domains (bottom). Each row represents one domain. Intergenic peaks were defined as >1.5 kb away from a promoter and with no overlapping repeat. **b**, Heatmap showing the number of peaks from **a** that overlap with the indicated chromatin signatures, defined as per Daugherty and colleagues^47^. **c**, Enrichment of TF motifs (log_2_-transformed) in the intergenic peaks from **a** versus genome-wide occurrence (log_2_-transformed). Enriched motifs (log_2_ enrichment > 0.5 and log_2_ abundance > 8) are colour-coded according to TF(s). *Enrichment of a scrambled motif. These motifs characterize enhancers in worms^47,48,52^. **d**, H3K9me3 (left) and H3K27ac (right) ChIC-seq enrichment (normalized to the MNAse control) at newly accessible intergenic peaks (**a**) in WT and *met-2*- and *met-2 set-25*-mutant muscle. Data points represent 10 kb bins over intergenic peaks. Boxplots show the median (horizontal line), 25th to 75th percentiles (boxes), and 90% (whiskers) of the group. ****P < 2.2 × 10^−12^; two-way analysis of variance, followed by a post-hoc test. **e**, Reporter constructs containing a minimal promoter driving GFP (nucleolar localization tag) with either no upstream sequence (control) or the *nhr-25*-associated enhancer (Enhancer reporter) as previously described^47^ (top). Representative images of GFP in the body region of WT and *met-2 set-25*-mutant animals (middle). Cells with GFP signal are highlighted with yellow arrows, all other signal is intestinal autofluorescence. Scale bar, 5 µm. Level of GFP expression as the proportion of worms with ≥5 GFP^+^ cells, excluding the head and tail (right). Control, *n* = 30 (WT) and 19 (*met-2 set-25*) worms; enhancer, *n* = 47 (WT) and 43 (*met-2 set-25*) worms from three independent biological replicates. Statistical significance was determined using a two-tailed *t*-test. **f**, Continuous deposition of H3K9me2 by MET-2 represses tissue-specific genes and enhancers (left). MET-2 activity is usually followed by SET-25, such that genes accumulate H3K9me3. In the absence of MET-2, genes that are derepressed require specific TFs (right). Perinuclear H3K9me-marked genes seem to be refractory to chromatin decompaction despite activation in a *met-2 set-25* mutant, whereas genes that gain both chromatin accessibility and transcriptional activity tend to localize internally. Unlike genes, enhancers can be repressed by either MET-2 or SET-25. Source data

**Extended Data Fig. 8:**
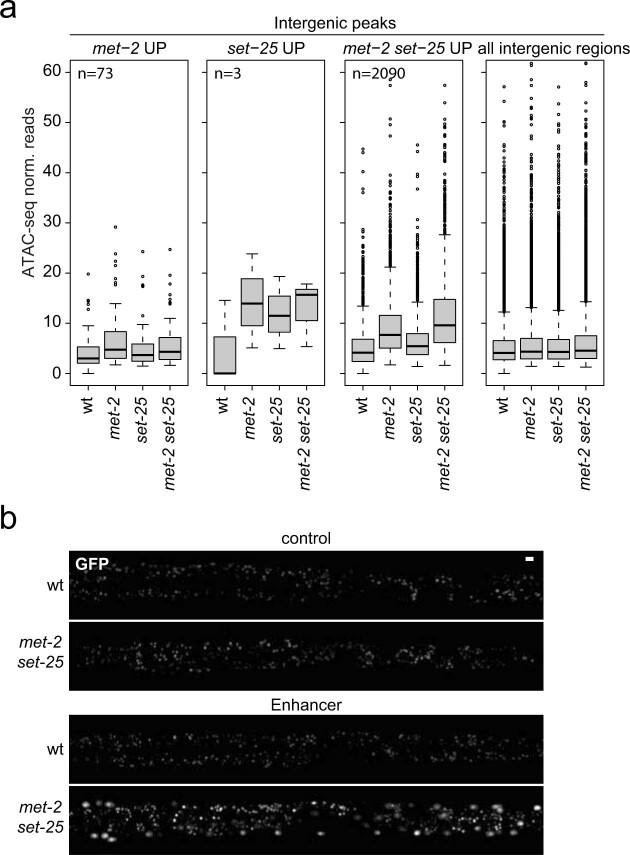
Characteristics of intergenic ATAC-seq peaks in HMT mutant muscle. a) The normalized ATAC-seq reads from muscle cells of indicated genotypes are plotted, as boxplots, for all intergenic peaks, or for those that gain accessibility in the indicated genotype. Boxplots show median, boxes 50% and whiskers 90% of the group. N = 3 biological independent replicas. b) Representative images of body region from wt or *met-2 set-25* worms expressing the control or enhancer reporter (Fig. 7e). Worms were straightened in ImageJ. Scale bar, 10 µm. The experiment has been performed for three times with similar results, n pooled worms analysed = wt control, 30; met-2 set-25 control, 19; wt enhancer, 47; met-2 set-25 enhancer, 43.

The presence of enhancer-linked TF motifs, coupled with the fact that Group C sites gain both H3K27ac and ATAC sensitivity following the loss of H3K9me (Fig. 6e), suggested that H3K9me might be limiting enhancer activity in WT tissues. To separate enhancer elements from overlapping promoters, we analysed the intergenic ATAC-seq peaks that gained accessibility in the *met-2 set-25*-mutant muscle (2,090; Fig. 6c). By comparing ATAC-seq accessibility at these intergenic peaks, we observed that *met-2* and *set-25* ablations were synergistic and these peaks were absent in WT muscle, where they showed baseline accessibility (Fig. 7a and Extended Data Fig. 8a). We examined other genomic features in the modENCODE datasets from whole-worm lysates^47^ for correlation with these regions. The strongest correlation for these intergenic peaks was with larval-specific enhancers (Fig. 7b). Moreover, these peaks were enriched for motifs for EOR-1, DAF-12/NHR-48 and ZTF-3 TF, which typically bind enhancer elements (compare Fig. 7c with Extended Data Fig. 7d). As expected, they were highly enriched for H3K9me3 in WT muscle, and this mark was partially maintained in the *met-2* mutant but not in the *met-2 set-25* double mutant (Fig. 7d). Moreover, the level of H3K27ac, which characterizes active enhancers^52^, showed a partial increase at these sites in *met-2*-mutant and was strongly elevated in *met-2 set-25* muscle cells (Fig. 7d). This suggests that enhancers, like promoters, are masked by H3K9me3 and can be targeted by both MET-2 and SET-25 redundantly.

Once again, to prove that H3K9me represses enhancer activity as it does gene expression, we made use of a reporter strain containing a transgenic array bearing a minimal promoter driving *gfp* with or without an upstream enhancer sequence that had been previously identified in proximity to the gene *nhr-25* (ref. ^47^). In the absence of the upstream enhancer, the loss of H3K9me had no effect on GFP expression within the body region of L3 larvae. However, when the enhancer element was included, cells in the *met-2 set-25* double mutant had strongly increased GFP expression (Fig. 7e and Extended Data Fig. 8b). Together, we conclude that H3K9me2/me3, deposited by either MET-2 or SET-25, or both, can limit not only promoter but also enhancer accessibility and activity.

## Discussion

We demonstrate an important function of H3K9me2/me3 in the silencing of cell type-specific genes in differentiated tissues in *C eleganst*. We found that following the loss of H3K9me3 (that is, in the *set-25* deletion), H3K9me2 serves to maintain gene repression in differentiated cells, highlighting a redundancy that serves to safeguard chromatin-mediated gene silencing. Even in the *met-2 set-25* double mutant, which loses both H3K9me2 and H3K9me3, misexpression of genes in differentiated tissues was dependent on the availability of appropriate TFs (Fig. 5). Given the developmental changes in the H3K9me pattern (Fig. 2), we propose that H3K9me reinforces differentiation-linked transcriptional programmes. In other words, tissue-specific target restriction by H3K9me, on both enhancers and promoters, allows TFs to promote distinct expression programmes at different stages of development and/or in different tissues. We confirm that potential binding sites of the conserved TF CEH-48 are occluded in at least three derepressed gene promoters in WT but not *met-2 set-25* larvae; importantly, CEH-48 is necessary for the misexpression of genes that lose H3K9me in muscle. Finally, loss of the TFs HLH-25 and HLH-28/-29, which also drive gene derepression in the *met-2 set-25*-mutant muscle, have pleiotropic defects in multiple tissues, including germline, in otherwise WT worms^38,54^. Our data thus argue that tissue-specific H3K9 methylation restricts the range of targets a factor can bind.

The role of H3K9me in gene silencing seems to not be linked to its function at REs, as we found no preferential proximity between the derepressed genes and specific repeat families. Only 26% of the 331 derepressed genes had REs within 1 kb of the corresponding transcriptional start site compared with 34% for all genes. Nonetheless, we do not exclude that individual REs could influence transcription at some loci. Even if REs were to help target HMTs to genes, TF binding is still likely to be necessary for derepression in *met-2 set-25*-mutant worms^53^.

Although the loss of H3K9me led to the misexpression of tissue-specific and germline genes, it is not essential for organismal development per se, as 88% of the *met-2 set-25* embryos form adult worms^3^. Nonetheless, we see that muscle integrity and function are impaired in the mutant. Whether such defects were caused by a specific misexpressed protein(s) or dysfunction due to imbalanced transcriptional homeostasis is not yet clear.

Surprisingly, although H3K9me seemed to prevent TF binding, a gain in accessibility as measured by ATAC-seq was neither required nor sufficient for gene derepression. One source of this discrepancy arises from the fact that enhancers can lose H3K9me and gain H3K27ac without activating the immediately adjacent genes (for example, Group C). However, approximately 80% of the genes derepressed in the *met-2 set-25* mutant (Group A) showed little change in promoter accessibility. At the genes that gain both accessibility and expression (Group B), an analysis of TF motifs indicated that chromatin decompaction may well be a secondary effect of TF binding, as the TFs recruit additional complexes that in turn regulate chromatin opening. Notably, the bZIP TFs that co-map with Group B loci as well as Onecut1/2, the CEH-48 homologue, recruit the CBP/p300 histone acetyltransferase, and EOR-1 cooperates with the SWI/SNF chromatin remodeler^51,54–57^. Thus, depending on the chromatin factors that are recruited, different TFs may trigger different degrees of chromatin opening (Fig. 7f).

Group B genes are further distinct in that they are not enriched at the nuclear periphery, which is in contrast to the majority of H3K9me3-marked loci^13,58^. This would argue that although subnuclear positioning is not the crucial determinant of gene expression^59^, it may determine whether or not a promoter is susceptible to decondensation following the loss of H3K9me. Alternatively, it may be that different combinations of TFs are responsible both for the gene position and its response to a loss of H3K9me. The transient opening of a promoter that allows transcription near the nuclear envelope may allow a derepressed gene to remain ATAC-resistant. Consistent with the idea that subnuclear positioning differentiates levels of accessibility, aberrant bZIP TF activity can induce the relocation of a heterochromatic array towards the nuclear interior, in a manner dependent on the HAT p300/CBP^50^. In summary, our study shows that H3K9me, like DNA methylation^60^, can restrict TF activity at promoters and enhancers and that it does this primarily at tissue-specific genes whose repression is required for tissue robustness.

## Methods

### Constructs and strains

The worm strains used in this study are listed in Supplementary Table 1.

To generate ubiquitously expressed TIR1, the eft-3 promoter was subcloned by Gibson cloning from the plasmid pIK130 into a Mos1-mediated single-copy insertion-compatible plasmid containing *TIR-1* and the *tbb-2* 3′ untranslated region. This new construct was inserted in the ttTi5605 locus on chromosome 2 by Mos1-mediated single-copy insertion^61^. Endogenously tagged *met-2*, *gw5(met-2::linker::degron::3×FLAG::TEV::NeonGreen)*, was generated using clustered regularly interspaced short palindromic repeats strategy of the Dokshin and colleagues^62^. Briefly, a *met-2*-specific (GTAACTTTTCAGCAAACGGC) crRNA (Horizon Discovery) was injected along with recombinant Cas9 (Lubio Science), trcRNA (Lubio Science) and a PCR template containing a single-stranded overhang with *met-2* homology and the *linker::degron::3×FLAG::TEV::NeonGreen* cassette, amplified from the pIK369 plasmid.

Except for RNAi experiments, worms were grown at 20 °C on NGM plates and fed OP50 bacteria. For the auxin treatments, the worms were placed on NGM plates containing 1 mM auxin for the indicated times. The RNAi experiments were performed as described previously^63,64^ using NGM plates (1 mM IPTG and 100 µg ml^−1^ carbenicillin), which were prepared with the appropriate RNAi-expressing bacteria and allowed to dry. Synchronized L1s were seeded on the RNAi and either L1 or L3 larvae (as indicated) of the following generation were processed for RNA-seq or microscopy (Fig. 5 and Extended Data Fig. 6).

### Phalloidin staining

Phalloidin staining of muscle fibres and scoring of muscle defects were performed as described previously^65^. Briefly, synchronized L1 larvae were grown to the young adult stage (before the appearance of eggs), and collected and washed with M9 buffer. The worms were then transferred to 1.5 ml low-bind tubes (Eppendorf) and fixed with 4% formaldehyde (Thermo Fischer) in 0.1 M Na_2_HPO4 for 15 min. The worms were pelleted, permeabilized in 500 μl of pre-chilled acetone (5 min at −20 °C) and then washed three times with PBS-TG (PBS, 0.5% Triton X-100 and 30 mM glycine). Rhodamine phalloidin (500 μl; R415, Life Technologies; 1:250 in PBS-TG) was added to the worms for 30 min at room temperature with rotation. The worms were washed three times, settled on poly-l-lysine-coated slides and mounted with ProLong Gold (P36930, Thermo Fischer).

### Microscopy and image analysis

Animal morphology images were taken at 20 °C using a Leica M205 FA microscope. All fluorescent microscopy images and movies for the swimming assay were taken at 20 °C on spinning-disk multipoint confocal microscopes using the VisiView software (Visitron): (1) AxioImager M1 with Yokogawa CSU-X1 scan head, A plan-NEOFLUAR ×100/1.45 oil, Rolera Thunder Back Illuminated EM-CCD (Q Imaging) and VisiView v.4.4.0.14; (2) Nikon Ti2-E Eclipse with Yokagawa CSU W1 scan head, CFI P-Apo Lambda ×60/1.4 oil, iXon-Ultra-888 Back illuminated EM-CCD (Andor) and VisiView v.4.5.0.10; and (3) Zeiss AxioObserver7 with Yokagawa CSU W1 scan head, EC Plan-Neofluar ×40/1.3 oil, Prime 95B sCMOS (Photometrics) and VisiView v.4.2.0.6. The GFP and NeonGreen, and RFP, mCherry and Rhodamine fluorophores were excited using Toptica iBeam Smart 488 nm and 561 nm lasers, respectively. For live microscopy, worms were collected off plates in M9 buffer and placed on 2% agarose pads. To enrich for L1 larvae, plates containing gravid adults were washed to remove adults and larvae, and newly hatched L1 worms were collected after 3–5 h by washing with M9. To enrich for L3 larvae, gravid adults were seeded for about 1 h on plates and then removed; semi-synchronous L3 larvae were collected after approximately 38–40 h by washing with M9.

Quantification of the GFP–LacI and PGL-1::GFP intensity in larval tissues of RNAi-treated worms was performed using the Fiji/ImageJ software. For PGL-1::GFP, the mean fluorescence intensity was measured in an area with representative signal and in the focal plane of each cell. For GFP–LacI, the GFP intensity was blindly measured as specific tissues and nuclei were identified and the nuclear area selected based on the CEC-4::WmCherry signal.

To perform quantification of the GFP–LacI intensity, spot volume and spot distance from nuclear periphery in muscle and intestine nuclei, individual nuclei were cropped in Fiji and analysed separately using the KNIME Analytics Platform^66^. In summary, nuclei were detected using a seeded watershed segmentation on the EMR-1 (mCherry) channel. For foci detection, we used a Laplacian-of-Gaussian detector from TrackMate^67^ (fmiij2-plugins-0.2.5, 10.5281/zenodo.1173536) on the GFP–LacI (GFP) channel. The distance between the foci and the nuclear periphery was measured by computing a Euclidean distance map on the three-dimensional nucleus mask and measuring its intensity for the coordinates of each spot. Foci outside of a nucleus were ignored in the analysis. The ‘distance from periphery’ was plotted per condition using the R package ggplot2 (https://ggplot2.tidyverse.org/).

### Swimming assay

Worms were placed in flat-bottomed 96-well plates in M9 buffer and movies with a duration of 1 min were acquired at 20 °C. For analysis, the movies were slowed fivefold to enable counting of the number of head turns over the 1 min recording time.

### Western blotting

Pellets of thoroughly washed L2 worms were flash-frozen in liquid nitrogen and kept at −80 °C until processing. Thawed pellets were lysed using a Fast Prep 24-5 G benchtop homogenizer (MP Biomedicals) and 0.5 mm zirconia/silicon beads (BioSpec) in RIPA buffer in the presence of cOmplete EDTA-free protease inhibitors (Roche) and 1 mM dithiothreitol (DTT) at 4 °C. The lysates were treated with 5 μl benzonase (Sigma) for 1 h at 4 °C with end-over-end rotation. Total protein (10 µg) was separated on Bolt 4–12% bis-Tris plus gels (Thermo Fisher) and transferred to a PVDF membrane (Bio-Rad). The blots were blocked in PBS plus 0.5% Tween-20 with 5% powdered milk. The primary antibodies used were: 1:1,000 mouse anti-FLAG M2-horseradish peroxidase (HRP) conjugated (A8592, Sigma), 1:500 mouse anti-H3K9me2 MABI0317 (MBL)^68^ and 1:5,000 rabbit anti-MRG-1 (49130002, Novus Biologicals). The blots were treated with antibodies diluted in blocking buffer overnight at 4 °C. Except anti-FLAG, after three washes, blots were re-blocked and exposed to secondary antibodies coupled to HRP (goat anti–mouse IgG HRP (Jackson ImmunoResearch, 115-035-146) at a 1:10,000 dilution and goat anti–rabbit IgG HRP (Jackson Immuno Research, 111-035-144) at a 1:50,000 dilution). After three subsequent washes, ECL (Millipore) was applied and signal was detected using an Amersham Imager 600 (GE). MRG-1 was specifically chosen for a loading control because it is a euchromatic nuclear protein, its expression remains unchanged in the *met-2 set-25* mutant and we have a well-characterized antibody against MRG-1.

### Worm dissociation and cell sorting

Isolation of worm tissues for FACS sorting was performed as previously described^69^ with minor adjustments. Synchronized L1 worms (200,000) were seeded on 15 cm peptone-rich plates for 32 h at 20 °C before processing. The worms were collected and thoroughly washed in 15 ml Falcon tubes using M9 solution before being transferred into multiple 1.5 ml low-bind tubes to obtain a pellet of approximately 100 ml for each sample (Eppendorf). The worms were resuspended in 200 μl SDS-DTT solution (20 mM HEPES pH 8.0, 0.25% SDS, 200 mM DTT and 3% sucrose) and incubated for exactly 4 min at room temperature before being resuspended in 800 μl of egg buffer (25 mM HEPES pH 7.3, 118 mM NaCl, 48 mM KCl, 2 mM CaCl_2_ and 2 mM MgCl_2_ with an osmolarity of about 340 mOsm). The worms were washed five times with 1 ml of egg buffer. The worm pellets were then resuspended in 100 μl of 15 mg ml pronase E (Sigma), diluted in egg buffer and pellets from the same sample were pooled. The worms were then vigorously resuspended with a thinned-out Pasteur pipette until most were visibly dissociated. Digestion was stopped with 900 μl of 10% FBS (in M9), the cells were washed twice with 10% FBS, with centrifugation at 9,600*g* for 5 min at 4 °C. After washing, the cells were resuspended in 1 ml of cold M9 buffer and left to settle for approximately 30 min on ice. The supernatant was collected and filtered into sorting tubes (Becton Dickinson) using 30 µm cell filters (Sysmex) and processed for cell sorting or ChIC-seq.

The filtered cells were kept on ice until sorting; 1 μl DRAQ7 (BioStatus) was added to the samples immediately before sorting to exclude dead cells. The cells were sorted into 1.5 ml low-bind tubes (Eppendorf) following a similar strategy using either a BD FACSAria (Becton Dickinson) or MA900 (Sony) cell sorter. For RNA-seq, approximately 25–50 × 10^3^ muscle cells or approximately 1 × 10^4^ seam cells per genotype per experiment (three replicas each) were sorted directly into 200 μl of lysis buffer (Norgen) and processed. For ATAC-seq, 50 × 10^3^ muscle cells per condition per experiment (three replicas each) were sorted into 200 μl M9 solution and processed. For ChIC-seq, see the ‘ChIC-seq’ section.

### RNA-seq

For isolated tissues, RNA was purified from lysed muscle or seam cells following the instructions of the Norgen single cell RNA purification kit (51800, Norgen Biotek). Libraries were produced using a Smart-Seq2 mRNA sequencing kit (Illumina). For L1 RNA-seq, worms were removed and thoroughly washed from RNAi plates using M9 solution. The worm pellet was resuspended in TRIzol, flash-frozen in liquid nitrogen and then freeze-cracked four times; the RNA was extracted by phenol–chloroform extraction, followed by isopropanol precipitation. The ribosomal RNA was depleted using a Ribo-Zero gold kit (Epicentre) and libraries were produced using a Total RNA sequencing TruSeq kit (Illumina). Equimolar amounts of indexed libraries were pooled and sequenced on a HiSeq 2500 system (Illumina). For data collection and conversion to fastq format, RTA 1.18.64 (HiSeq2500), RTA 2.4.11 (NextSeqS00) and bcl2fastq2 v2.17 were used.

The reads were analysed as described previously^70^. The reads were aligned to the *C. elegans* genome (ce10) using the R package QuasR v1.22.0, (www.bioconductor.org/packages/2.12/bioc/html/QuasR.html)^71^. The command ‘proj < −qAlign (‘samples.txt’, ‘BSgenome. Celegans.UCSC.ce10’, ‘splicedAlignment = TRUE’)’ instructs hisat2 (ref. ^72^) to align using default parameters, considering unique reads for genes and genome-wide distribution. Count tables of reads mapping within annotated exons in WormBase (WS220) were constructed using the qCount function of the QuasR package to score the number of reads in each window (qCount(proj,GRange_object, orientation = ‘same’)) and normalized by division by the total number of reads in each library and multiplied by the average library size. Transformation into the log_2_ space was performed after the addition of a pseudo count of eight to minimize large changes in abundance FC caused by low count numbers. The EdgeR package v.3.24 was applied to select genes with differential transcript abundances between indicated the genotypes (contrasts) based on the FDR for genes. Annotation of tissue and cell-type expression is based on annotation tables provided by the tissue atlas^73^.

### ChIC-seq

Filtered cells were pelleted, resuspended in 1 ml M9 solution and then fixed by slowly adding 2.33 ml of cold (−20 °C) EtOH (100%) while vortexing the cells. The cells were left at −20 °C for 1 h, centrifuged and washed with wash buffer 1 (WB1; 20 mM HEPES pH 7.5, 150 mM NaCl, 0.46 mM spermidine, 0.05% Tween-20, 1×cOmplete protease inhibitor and 2 mM EDTA). The washed cells were resuspended in WB1 + 10% dimethylsulfoxide and stored at −80 °C until ready to process.

The cells were thawed on ice (all subsequent steps were performed on ice) and washed once with WB1. The cells were then counted and diluted in WB1 to approximately 5 × 10^5^ cells in 200 μl. For each antibody and mutant, 200 μl of cells was transferred to 0.5 ml low-bind tubes (Eppendorf). Primary antibodies (200 μl), diluted to 2× in WB1, were then added to the cells and incubated overnight on a rotator at 4 °C. The antibodies used were to: H3K9me3 (1:2,000; MABI0318, MBL)^68^, H3K9me2 (1:5,000; MABI0317, MBL)^68^ and H3K27ac (1:5,000; ab177178, Abcam). The cells were washed once with wash buffer 2 (WB1 with no EDTA), then resuspended in 500 µL of WB2 containing 1 ng ml^−1^ ProteinA-MNase (recombinant) and 5 µg ml^−1^ Hoechst 33258, and incubated on a rotator for 1 h at 4 °C. The cells were then washed twice with 500 μl of wash buffer 2 (WB1 with no EDTA) and filtered into sorting tubes with a final volume of approximately 500 μl WB2. For each sample, approximately 5 × 10^3^ cells were sorted into 5 μl of wash buffer 3 (WB1 with no EDTA and no protease inhibitor). Hoechst was used as a sorting marker for gating fixed/lysed cells.

After sorting, MNase was activated by adding 10 μl wash buffer 3 containing 4 mM CaCl_2_ for 30 min on ice. The reaction was then stopped with the addition of 20 μl Stop solution (40 mM EGTA, 1.5% NP-40 and 2 mg ml^−1^ proteinase K) and the proteins were digested (65 °C for 6 h, 80 °C for 20 min and hold at 4 °C).

DNA fragments were blunt ended by adding 30 μl of blunt end mix (2.5 U Klenow large fragment, 5 U T4-PNK, 0.4 mM dNTPs, 2.3 mM ATP, 1.7 mM MgCl_2_, 7 μl PNK buffer 10×, 0.24 ng ml^−1^ BSA and 2.5% PEG8000) and incubating for 30 min at 37 °C, followed by 20 min at 75 °C for enzyme inactivation. The fragments were A-tailed by adding 30 μl tail mix (1 U AmpliTaq 360, 0.7 mM dATP, 167 mM KCl, 0.1 ng ml^−1^ BSA and 2.5% PEG8000) and incubation at 72 °C for 15 min. The fragments were further ligated to T-tail-containing adaptors with the sequence: forward strand, *GGTGAT*GCCGGTAATACGACTCACTATAGG*GAGTTCTACAGTCCGACGATC*NNNACACACTAT and reverse strand, /5Phos/TAGTGTGTNNN *GATCGTCGGACTGTAGAACTC*CCTATAGTGAGTCGTATTACCGGC*GAGCTT*. In the forward strand, the first six italic basepairs form a fork, the underlined basepairs after that are a T7 promoter, the following italic bases are the binding site (RA5) for the TruSeq Small RNA indexing primers (RPIx) and the underlined three random base pairs represent the unique molecular identifier. Ligation was performed by adding 40 μl ligation mix (1.25 µM adaptor, 2,000 U T4 ligase, 17.5 mM MgCl_2_, 52.5 mM Tris pH 7.5, 26.25 mM DTT, 1.75 mM ATP, 0.1 ng ml^−1^ BSA and 2.5% PEG8000) and incubating for 20 min at 4 °C, followed by 16 h at 16 °C. The ligase was inactivated by incubation for 10 min at 65 °C.

The ligation reactions were cleaned with Ampure XP beads (Beckman Coulter) at a 0.8× beads-to-sample ratio and resuspended in 8 μl clean water. The cleaned fragments were amplified by adding 12 μl of MEGAscript T7 transcription kit and incubation for 12 h at 37 °C. The template DNA was removed by the addition of 2 μl TurboDNAse (IVT kit) and incubation for 15 min at 37 °C. The produced RNA was further purified using RNA clean XP beads (Beckman Coulter) at a 0.8× ratio, followed by RNA fragmentation for 2 min at 94 °C. After another bead clean-up, 40% (5 μl) of the RNA was primed for reverse transcription by adding 1.5 μl of 3.3 mM dNTPs and 13.3 µM random hexamer RT primer (GCCTTGGCACCCGAGAATTCCANNNNNN), and hybridizing it by incubation at 65 °C for 5 min, followed by direct cool down on ice. Reverse transcription was performed by the further addition of 4 μl RT mix (2.5×first strand buffer, 25 mM DTT, 20 U RNAseOUT and 100 U SuperscriptII) and incubation at 25 °C for 10 min, followed by 1 h at 42 °C. The single-stranded DNA was purified through incubation with 5 µg or 25 U RNAseA for 30 min at 37 °C and PCR amplification to add the Illumina smallRNA barcodes and handles. Next, 40 μl of PCR mix (3.1×NEBNext Ultra II Q5 master mix (5×stock), 0.5 µM RP1 and 0.5 µM RPIx primers) was added. The number of PCR cycles performed were dependent on the abundance of the histone modification assayed, and the PCR products were cleaned twice using Ampure XP beads at a 0.8× ratio. The final libraries were eluted in 7 μl nuclease-free water and the abundance and quality were assessed using the QUBIT and Bioanalyzer systems.

### ChIC-seq analysis

Equimolar amounts of indexed libraries were pooled and sequenced on a HiSeq 2500 (Illumina). Adaptors were trimmed using Trimmomatic 0.39 (http://www.usadellab.org/cms/?page=trimmomatic)^74^ with parameters set to: CROP:75 HEADCROP:12. Reads were aligned to the ce10 genome assembly using bowtie2 (http://bowtie-bio.sourceforge.net/bowtie2/index.shtml)^75^ with alignment parameters set to sensitive. The reads were then deduplicated using the deduplicate_bismark function in the Bismark program (https://github.com/FelixKrueger/Bismark)^76^. The read density was calculated by tiling the genome into 500 bp non-overlapping windows and using the qCount function of the QuasR package to quantify the number of reads in each window (qCount(proj,GRange_object,orientation = ‘any’)). H3K9me-positive domains were determined as regions with a consecutive enrichment of H3K9me2 and/or H3K9me3 over the input. For gene quantification, gene annotation from WormBase was used (WS220). Quantitation for each gene was performed by counting the reads overlapping the exons. Genome annotation was based on the BSgenome.Celegans.UCSC.ce10 package (https://bioconductor.org/packages/release/data/annotation/html/BSgenome.Celegans.UCSC.ce10.html). Differences in read depths between samples were normalized by dividing each sample by the total reads and multiplying by the average library size. The various count tables used throughout this study were normalized to the total genome count. The log_2_-transformed expression levels were determined after the addition of a pseudo count of eight (*y* = log_2_(*x* + 8)) to minimize large changes in FC caused by low count numbers. To control for unspecific activity of the MNase enzyme, results are displayed as: (mean enrichment of antibody + MNase–protein A-treated sample) ÷ MNase–protein A-treated sample. Replicate experiments were combined by averaging the enrichment values. Annotation of tissue and cell-type expression is based on annotation tables provided by the tissue atlas^73^. For comparison of chromosome arms versus centres, the following border coordinates were used: chromosome 1, 3745632 and 10809938; chromosome 2, 4708341 and 11877168; chromosome 3, 3508994 and 9947268; chromosome 4, 7317812 and 12176625; chromosome 5, 8125434 and 13849337; and chromosome X, 41919369. Pairwise Wilcoxon rank-test values were calculated in R.

### ChIP–qPCR

Synchronized L1 animals were seeded on peptone-rich plates and L3 animals were harvested 36 h later. The worms were washed in M9 solution until clear. The worm pellet was then resuspended in 10 ml of M9 containing 2% formaldehyde and fixed for 30 min at room temperature on a rocker. Fixation was stopped with 500 μl of 2.5 M glycine for 5 min at room temperature on a rocker. The worms were then washed twice with M9. The worm pellet was resuspended in 1 ml of FA buffer (50 mM HEPES pH 7.5, 1 mM EDTA, 1% Triton X-100, 0.1% Na-deoxycholate and 150 mM NaCl) containing 1% sarcosyl, transferred into bead-beater tubes with 200 μl of 0.5 mm zirconia/silicon beads and the worms were lysed in a Fast Prep 24-5 G benchtop homogenizer. The recovered supernatant was transferred into 15 ml polystyrene tubes and sonicated (30 cycles; 30 s on/off at 4 °C) using the Bioruptor plus sonicator (Diagenode). The samples were transferred to DNA LoBind tubes (Eppendorf), were centrifuged at maximum speed for 5 min at 4 °C, and the supernatant (containing chromatin) was collected. For each sample, 100 μg of chromatin was diluted to 600 μl in FA buffer, and incubated overnight on a rocker at 4 °C with 5 μl of GFP-Trap magnetic particles (ChromoTek). The magnetic particles were washed with the following buffers: 3 × 5 min with FA buffer, 5 min with FA buffer containing 1 M NaCl, 10 min with FA buffer containing 500 μl NaCl, 5 min with TEL buffer (0.25 M LiCl, 1% NP-40, 1% Na-deoxycholate, 1 mM EDTA and 10 mM Tris–HCl pH 8) and 2 × 5 min with TE. The complexes were eluted with 100 μl of elution buffer (1% sarcosyl in TE with 250 mM NaCl) at 65 °C with shaking at 1,100 r.p.m. for 15 min. Cross-links were reversed overnight at 65 °C, followed by addition of 20 μg proteinase K and further incubation for 2 h at 65 °C. The DNA was then purified using AMPure XP beads at a 0.8× ratio.

### ATAC-seq

ATAC-seq was performed on sorted muscle cells following the Omni-ATAC protocol^77^. Briefly, sorted cells were centrifuged for 10 min at 1,000*g* (4 °C) using a swinging-bucket rotor, and the supernatant was carefully removed to avoid disturbing the cell pellet. The cells were lysed for 3 min in 50 μl of cold resuspension buffer (10 mM Tris–HCl pH 7.4, 10 mM NaCl and 3 mM MgCl_2_) containing 0.1% NP-40, 0.1% Tween-20 and 0.01% digitonin. Lysis was stopped with 1 ml of cold resuspension buffer containing 0.1% Tween-20, and the nuclei were pelleted for 10 min at 1,000*g* (4 °C). The supernatant was carefully removed and the nuclei were resuspended in 50 μl of transposition mix (25 μl 2×TD buffer, 2.5 μl Tn5, 16.5 μl PBS, 0.5 μl of 1% digitonin, 0.5 μl of 10% Tween-20 and 5 μl double-distilled water) and incubated for 30 min at 37 °C on a thermomixer. The DNA was then purified using a Qiagen MinElute PCR purification kit according to the manufacturer’s instructions and eluted in 21 μl of elution buffer. Libraries were amplified as originally described^78^ and the final preparations were purified using a 1.6× ratio of AMPure XP beads. Sequencing was performed using NextSeq, with 75 cycles and paired-end reads.

### ATAC-seq analysis

The ATAC-seq reads were pre-processed following the ENCODE ATAC-seq pipeline (https://github.com/ENCODE-DCC/atac-seq-pipeline). Adaptors were trimmed using cutadapt (https://github.com/marcelm/cutadapt;^19^) and reads were aligned to the ce10 genome assembly using bowtie2 (http://bowtie-bio.sourceforge.net/bowtie2/index.shtml)^79^ with alignment parameters set to sensitive. The mapped reads were marked and removed using the MarkDuplicates function of Picard tools v.2.20.0 (https://broadinstitute.github.io/picard/). Paired-end reads were converted to single-end reads using bedtools 2.26 (https://github.com/arq5x/bedtools2)^80^ and the reads were shifted +4 bp and −5 bp for the positive and negative strands, respectively, to account for characteristics of the Tn5 enzyme. Peaks were called using MACS2 (ref. ^81^) with the following parameters: -g 93260000–nomodel–shift -100–extsize 200. Differential peaks among the genotypes were called using the EdgeR package v.3.24.The read density was calculated by tiling the genome into 500 bp non-overlapping windows or specifically at promoters (defined as 1 kb upstream and 100 bp downstream of the TSS) and using the qCount function of the QuasR package to quantify the number of reads in each window (qCount(proj,GRange_object,orientation = ‘any’)). Differences in read depths between samples were normalized by dividing each sample by the total number of reads and multiplying by the average library size. The various count tables used throughout this study were normalized to the total genome count. Definitions of characteristic chromatin domains was taken from^82^.

### TF-motif analysis

A compendium of TF weight matrices was downloaded (http://hugheslab.ccbr.utoronto.ca/supplementary-data/CeMotifs/) to examine the set in the file ‘TF_Information.txt’. These were used to scan the *C. elegans* ce10 genome using the matchPWM function in the Bioconductor package Biostrings. Only hits with a minimum score of ten were considered, unless the maximum obtainable score by the weight matrix was lower than ten. In that case, the maximum obtainable score was required. The resulting binding sites were then intersected with gene-promoter annotation (WS220; 1,500 bp 5′ and 500 bp 3′ of the TSS) to determine the number of sites for each TF and gene.

### Statistics and reproducibility

The experiments shown in this study were performed as 2–4 biologically independent experiments, as indicated in the figure legends, and no inconsistent results were observed. Data plotted as boxplots indicate the 25th and 75th percentiles, with the whiskers showing the minima and maxima (5th and 95th percentiles), black circles indicating the outliers and the horizontal line showing the median. Some data are plotted in bar graphs as the mean ± s.d, unless specified otherwise. If not stated otherwise statistical testing was performed using two-sided a Wilcoxon rank-sum test. The FDR index was calculated using the edgeR package (see Methods). Details of the particular statistical analyses used, precise *P* values, statistical significance, number of independent biological replicates and sample sizes for all of the graphs are indicated in the figures or figure legends. No data were excluded.

### Reporting Summary

Further information on research design is available in the Nature Research Reporting Summary linked to this article.

## Online content

Any methods, additional references, Nature Research reporting summaries, source data, extended data, supplementary information, acknowledgements, peer review information; details of author contributions and competing interests; and statements of data and code availability are available at 10.1038/s41556-021-00776-w.

## Supplementary information

Reporting Summary

Peer Review Information

Supplementary Tables 1–3 Supplementary Table 1. List of strains used in this study. Supplementary Table 2. List of primers used in this study. Supplementary Table 3. List of TF motifs identified in the *pgl-1* promoter (1,500 bp upstream and 500 bp downstream of the TSS). The number of motifs in the *pgl-1* promoter, along with the number of motifs genome-wide, are also indicated.

## Data Availability

All datasets from this study have been uploaded to the Gene Expression Omnibus (GEO) with the accession code of GSE167168. Previously published EMR-1 DamID and embryo RNA-seq/ChIP–seq data that were re-analysed here are available under the accession codes GSE136577 and SRP080806. All other data supporting the findings of this study are available from the corresponding author on reasonable request. Source data are provided with this paper.

## Source data

Source Data Fig. 3 Unprocessed western blots (related to Fig. 3c).
Source Data Fig. 3 Statistical source data.
Source Data Fig. 5 Statistical source data.
Source Data Fig. 7 Statistical source data.
Source Data Extended Data Fig. 1 Statistical source data.
Source Data Extended Data Fig. 6 Statistical source data.
