# Supplementary material for: H3K9me selectively blocks transcription factor activity and ensures differentiated tissue integrity
Source: Nat Cell Biol. 2021 Nov 4;23(11):1163–75. doi: 10.1038/s41556-021-00776-w (PMC8572725; doi:10.1038/s41556-021-00776-w)
Supplement: Supplementary file 1 — Reporting Summary [file 41556_2021_776_MOESM1_ESM.pdf]

nature portfolio

Corresponding author(s): Susan M. Gasser

Last updated by author(s): Sep 13, 2021

Reporting Summary

Nature Portfolio wishes to improve the reproducibility of the work that we publish. This form provides structure for consistency and transparency in reporting. For further information on Nature Portfolio policies, see our Editorial Policies and the Editorial Policy Checklist.

Statistics

For all statistical analyses, confirm that the following items are present in the figure legend, table legend, main text, or Methods section.

n/a	Confirmed	
☐	☒	The exact sample size (*n*) for each experimental group/condition, given as a discrete number and unit of measurement
☐	☒	A statement on whether measurements were taken from distinct samples or whether the same sample was measured repeatedly
☐	☒	The statistical test(s) used AND whether they are one- or two-sided<br>*Only common tests should be described solely by name; describe more complex techniques in the Methods section.*
☐	☒	A description of all covariates tested
☐	☒	A description of any assumptions or corrections, such as tests of normality and adjustment for multiple comparisons
☐	☒	A full description of the statistical parameters including central tendency (e.g. means) or other basic estimates (e.g. regression coefficient) AND variation (e.g. standard deviation) or associated estimates of uncertainty (e.g. confidence intervals)
☐	☒	For null hypothesis testing, the test statistic (e.g. *F*, *t*, *r*) with confidence intervals, effect sizes, degrees of freedom and *P* value noted<br>*Give P values as exact values whenever suitable.*
☒	☐	For Bayesian analysis, information on the choice of priors and Markov chain Monte Carlo settings
☒	☐	For hierarchical and complex designs, identification of the appropriate level for tests and full reporting of outcomes
☐	☒	Estimates of effect sizes (e.g. Cohen's *d*, Pearson's *r*), indicating how they were calculated

Our web collection on statistics for biologists contains articles on many of the points above.

Software and code

Policy information about availability of computer code

Data collection	For data collection and conversion to fastq format RTA 1.18.64 (HiSeq2500), RTA 2.4.11 (NextSeq500) and bcl2fastq2 v2.17 were used. Images were acquired on spinning disk multipoint confocal microscopes using Visiview software (Visitron): (1) AxioImager M1 with Yokogawa CSU-X1 scan head, A plan-NEOFLUAR 100×/1.45 oil, Rolera Thunder Back Illuminated EM-CCD (Q Imaging) and VisiView v.4.4.0.14. (2) Nikon Ti2-E Eclipse with Yokagawa CSU W1 scan head, CFI P-Apo Lambda 60x/1.4 oil, iXon-Ultra-888 Back illuminated EM-CCD (Andor) and Visiview v4.5.0.10. GFP/NeonGreen and RFP/mCherry/Rhodamine flurophores were excited using a Toptica iBeam Smart 488-nm and 561-nm lasers, respectively.
Data analysis	R package QuasR v1.22.0, EdgeR package v3.24, BSgenome.Celegans.UCSC.ce10 v1.0 package, Trimmomatic v0.39, bowtie2 v2.3.5.1, Bismark program v0.22.3 (https://github.com/FelixKrueger/Bismark), bedtools v2.26, pickard tools v2.20.0 ,ATACseq reads were preprocessed following the ENCODE ATACseq pipeline (https://github.com/ENCODE-DCC/atac-seq-pipeline). plots were created using ggplot2 v3.3.5, Microscopy images were analysed using the Fiji/ImageJ v1.53c and the KNIME Analytics Platform v4.3.3 software, with TrackMate v0.2.5. FACS analysis was performed using BD FACSDiva v8.0.1, MA900 Software, and FlowJo 10.6.2.

For manuscripts utilizing custom algorithms or software that are central to the research but not yet described in published literature, software must be made available to editors and reviewers. We strongly encourage code deposition in a community repository (e.g. GitHub). See the Nature Portfolio guidelines for submitting code & software for further information.

Data

Policy information about availability of data

All manuscripts must include a data availability statement. This statement should provide the following information, where applicable:

- Accession codes, unique identifiers, or web links for publicly available datasets
- A description of any restrictions on data availability
- For clinical datasets or third party data, please ensure that the statement adheres to our policy

All datasets from this study have been uploaded to the Gene Expression Omnibus (GEO) with the accession code of GSE167168 and GSE136577 , or the Sequence Read Archive (SRA) with the accession code of SRP080806

Field-specific reporting

Please select the one below that is the best fit for your research. If you are not sure, read the appropriate sections before making your selection.

☒ Life sciences ☐ Behavioural & social sciences ☐ Ecological, evolutionary & environmental sciences

For a reference copy of the document with all sections, see nature.com/documents/nr-reporting-summary-flat.pdf

Life sciences study design

All studies must disclose on these points even when the disclosure is negative.

Sample size	No statistical methods were used to predetermine sample size. All experiments were conducted with at least two independent biological replicate cell lines. For full list of strains used in this study see supplementary information.
Data exclusions	no data was excluded.
Replication	Experimental data was reliably reproduced. Each experiment was performed at least twice
Randomization	samples were allocated to groups according to genotype
Blinding	No blinding. Group allocation according to genotype was done before data collection.

Reporting for specific materials, systems and methods

We require information from authors about some types of materials, experimental systems and methods used in many studies. Here, indicate whether each material, system or method listed is relevant to your study. If you are not sure if a list item applies to your research, read the appropriate section before selecting a response.

Materials & experimental systems

n/a	Involved in the study
☐	☒ Antibodies
☒	☐ Eukaryotic cell lines
☒	☐ Palaeontology and archaeology
☐	☒ Animals and other organisms
☒	☐ Human research participants
☒	☐ Clinical data
☒	☐ Dual use research of concern

Methods

n/a	Involved in the study
☐	☒ ChIP-seq
☐	☒ Flow cytometry
☒	☐ MRI-based neuroimaging

Antibodies

Antibodies used	mouse anti H3K9me3 (MABI0318 (MBL; Kimura et al., 2008), mouse anti H3K9me2 (MABI0317 (MBL; Kimura et al., 2008) and recombinant anti H3K27ac (ab177178), mouse anti-FLAG M2-HRP conjucated (A8592, Sigma), rabbit anti-MRG-1 (#49130002, Novus Biologicals), recombinant anti-H3K27ac (ab177178), goat anti-mouse IgG HRP (Jackson ImmunoResearch 115-035-146), goat rabbit IgG HRP (Jackson Immuno Research 111-035-144)
Validation	For mouse anti H3K9me3 and mouse anti H3K9me2 see: Kimura H, Hayashi-Takanaka Y, Goto Y, Takizawa N, Nozaki N. The organization of histone H3 modifications as revealed by a panel of specific monoclonal antibodies. Cell Struct Funct. 2008;33(1):61-73. doi:10.1247/csf.07035 and Zeller P, Padeken J, van Schendel R, Kalck V, Tijsterman M, Gasser SM. Histone H3K9 methylation is dispensable for Caenorhabditis elegans development but suppresses RNA:DNA hybrid-associated repeat instability. Nat Genet. 2016;48(11):1385-1395. doi:10.1038/ng.3672<br>H3K27ac (ab177178) was tested as on a peptide array, ChIP enrichment at known targets, immunostaing and WB on TSA treated

cells, for references see: https://www.abcam.com/histone-h3-acetyl-k27-antibody-ep16602-chip-grade-ab177178.html
The mouse anti-FLAG M2-HRP conjucated (A8592, Sigma) was tested against wt lysates (no FLAG tag) in WB and it is validated by Sigma and widely used in the research community https://www.sigmaaldrich.com/catalog/product/sigma/a8592?lang=de&region=CH
The anti-MRG-1 (#49130002, Novus Biologicals) was validated by Novus Biologicals in WB and Immunofluorescence against wild-type and MRG-1 deficient cells (https://www.novusbio.com/products/mrg-1-antibody_49130002#ReviewsSection)

Animals and other organisms

Policy information about studies involving animals; ARRIVE guidelines recommended for reporting animal research

Laboratory animals	The manuscript utilized Caenorhabditis elegans (variant Bristol) as non-vertebrate model organism. A list of specific strains is attached as Supplementary information. Animals used for this study were hermaphrodites. Ages/developmental stages are indicated in the manuscript (ranging from early embryos (<200 cells), to 1 day old adults)
Wild animals	this study did not use wild animals
Field-collected samples	this study did not use field-collected samples
Ethics oversight	As a non-vertebrate, C. elegans does not fall under the Directive 2010/63/EU of the European Parliament and of the Council of 22 September 2010 on the protection of animals used for scientific purposes: http://eur-lex.europa.eu/LexUriServ/LexUriServ.do?uri=OJ:L:2010:276:0033:0079:en:PDF

Note that full information on the approval of the study protocol must also be provided in the manuscript.

ChIP-seq

Data deposition

☒ Confirm that both raw and final processed data have been deposited in a public database such as GEO.

☒ Confirm that you have deposited or provided access to graph files (e.g. BED files) for the called peaks.

Data access links<br>*May remain private before publication.*	GSE167168; SRP080806; ChIPseq experiments were previously published in https://doi.org/10.1038/ng.3672
Files in database submission	raw files of H3K9me2 and H3K9me3 ChIPseq in early embryos and ChIC-seq in isolated muscle, tab file of enrichments over 500bp genome tiles.
Genome browser session<br>(e.g. UCSC)	no longer applicable

Methodology

Replicates	ChIPseq were performed in triplicates
Sequencing depth	All samples were sequenced paired end, reads are 50nt long.<br>sample sample total_reads unique_mapped_reads<br>wt_input_H3K9me3_rep1 54390246 46979366<br>wt_input_H3K9me3_rep2 59795570 51509114<br>wt_input_H3K9me2_rep1 47541520 39146914<br>wt_input_H3K9me2_rep2 47340436 39010602<br>wt_IP-H3K9me3_rep1 75269058 49636930<br>wt_IP-H3K9me3_rep2 76904620 48796670<br>wt_IP-H3K9me2_rep1 37307384 24931938<br>wt_IP-H3K9me2_rep2 37183678 24859832
Antibodies	mouse anti H3K9me3 (MABI0318 (MBL; Kimura et al., 2008), mouse anti H3K9me2 (MABI0317 (MBL; Kimura et al., 2008)
Peak calling parameters	no peak calling was performed
Data quality	Plotting log2 enrichments genome wide and per gene for each replicate against each other showed a good correlation, indicating reproducibility.
Software	R package Bioconductor is version 3.12, QuasR v1.22.0, EdgeR package v3.24, Trimmomatic v0.39, bowtie2, Bismark program (https://github.com/FelixKrueger/Bismark), ggplot2 v3.3.2

Flow Cytometry

Plots

Confirm that:

☒ The axis labels state the marker and fluorochrome used (e.g. CD4-FITC).

☒ The axis scales are clearly visible. Include numbers along axes only for bottom left plot of group (a 'group' is an analysis of identical markers).

☒ All plots are contour plots with outliers or pseudocolor plots.

☒ A numerical value for number of cells or percentage (with statistics) is provided.

Methodology

Sample preparation	Isolation of worm tissues for FACS sorting was performed as previously{Zhang, 2011 #47} with minor adjustments. 200,000 synchronized L1 worms were seeded on 15cm Peptone-rich plates for 32 hrs at 20oC before processing. Worms were collected and thoroughly washed in 15 mL falcon tubes using M9 solution, before being transferred into multiple 1.5 mL low-bind tubes in order to have an ~100mL pellet each (Eppendorf). Worms were resuspended in 200 µL of SDS-DTT solution (20 mM HEPES pH 8.0, 0.25% SDS, 200 mM DTT, 3% sucrose) and incubated for exactly 4 mins at RT, before being resuspended in 800 µL of egg buffer (25 mM HEPES pH 7.3, 118 mM NaCl, 48 mM KCl, 2 mM CaCl2, 2 mM MgCl2 with osmolarity ~340 mOsm). Worms were washed 5 times with 1 mL of egg buffer. Worm pellets were then resuspended in 100 µL of 15 mg/mL pronase E (Sigma), diluted in egg buffer, and pellets from the same sample were pooled. Worms were then vigorously resuspended using a thinned-out Pasteur-pipette until most worms were visibly dissociated. Digestion was then stopped with 900 µL of 10% FBS (in M9). Cells were then wash 2 times with 10% FBS, with centrifugation of 9,600 g for 5 mins at 4oC. After washes, cells were resuspended in 1 mL of cold M9 and left to settle for ~30 mins on ice. Supernatant was collected and filtered into sorting tubes (Becton Dickinson) using 30 µm cell filters (Sysmex) then processed for cell sorting or ChIC-seq. After filtering, cells were kept on ice until ready to sort. Immediately prior to sorting, 1 µL of DRAQ7 (BioStatus), was added to cells in order to exclude dead cells.
Instrument	BD FACSAria cell sorter (Becton Dickinson) and MA900 cell sorter (Sony)
Software	BD FACSDiva v8.0.1, MA900 Software, and FlowJo 10.6.2
Cell population abundance	For muscle cells, the sorted population was analyzed by flow cytometry to determine purity. This yielded >90% purity. Furthermore, the transcriptome from sorted muscle cells was compared with non-muscle (neg ative cell sort) and demonstrated a significant enrichment for muscle specific transcripts. (see Extended Data Fig. 2)
Gating strategy	For muscle cells, the cell population was first selected by gating on FSC-A/SSC-A, and doublets removed with FSC-H/FSC-W followed by SSC-H/SSC-W. Muscle cells were then selected as the myo-3p::RFP positive, DRAQ7 negative population.<br>For Seam cells, cell selection was done as for muscle, but DRAQ7 negative (alive) cells were selected, followed by gating for seam cells as SCMp::GFP, AJM-1::mCherry double positive.<br>For each experiment the positive gate was determined by including a negative (N2) control.

☒ Tick this box to confirm that a figure exemplifying the gating strategy is provided in the Supplementary Information.
