# Supplementary figures and images for: H3K9me selectively blocks transcription factor activity and ensures differentiated tissue integrity
Source: Nat Cell Biol. 2021 Nov 4;23(11):1163–75. doi: 10.1038/s41556-021-00776-w (PMC8572725; doi:10.1038/s41556-021-00776-w)

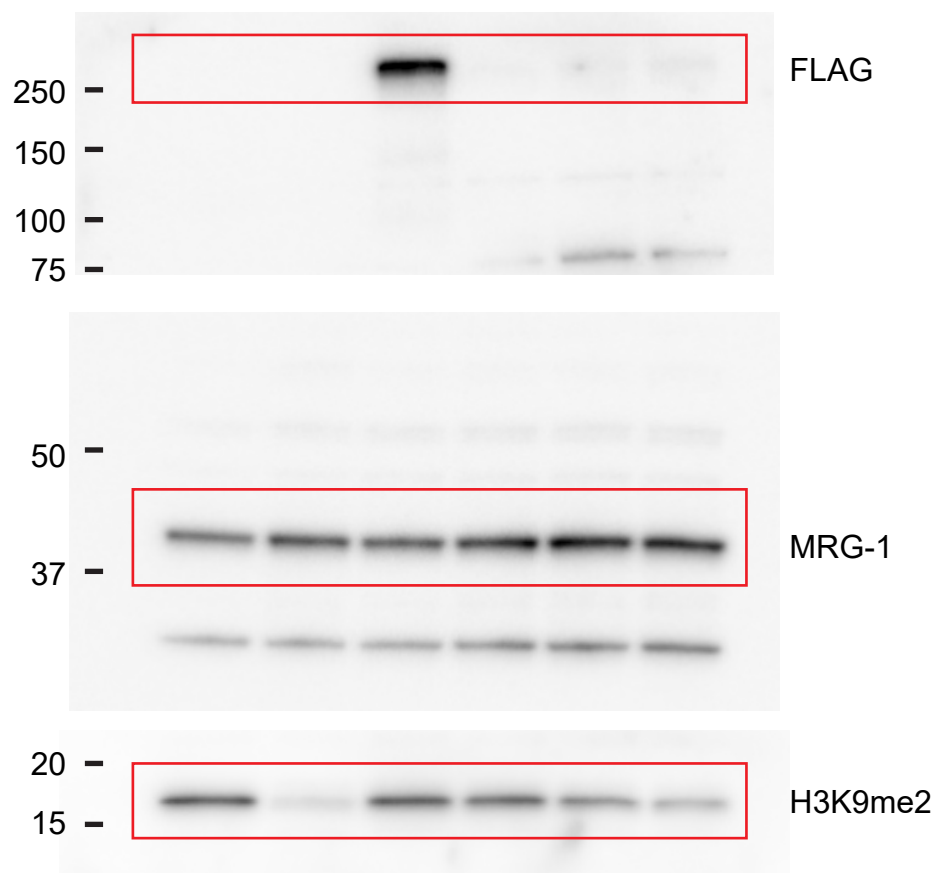

**Fig. 3c**

Supplement: Source Data Fig. 3 — Unprocessed western blots (related to Fig. 3c). [file 41556_2021_776_MOESM4_ESM.pdf]
