# Supplementary material for: H3K9me selectively blocks transcription factor activity and ensures differentiated tissue integrity
Source: Nat Cell Biol. 2021 Nov 4;23(11):1163–75. doi: 10.1038/s41556-021-00776-w (PMC8572725; doi:10.1038/s41556-021-00776-w)
Supplement: Supplementary file 2 — Peer Review Information [file 41556_2021_776_MOESM2_ESM.pdf]

natureresearch

Peer Review Information

Journal: Nature Cell Biology
Manuscript Title: H3K9me selectively blocks transcription factor activity and ensures differentiated tissue integrity
Corresponding author name(s): Professor Susan Gasser

Editorial Notes:

**Redactions – transferred manuscripts (mention of previous referee reports from elsewhere)**	This manuscript has been previously reviewed at another journal. This document only contains reviewer comments, rebuttal and decision letters for versions considered at Nature Cell Biology. Mentions of prior referee reports have been redacted.
**Redactions – transferred manuscripts (mention of the other journal)**	This manuscript has been previously reviewed at another journal. This document only contains reviewer comments, rebuttal and decision letters for versions considered at Nature Cell Biology. Mentions of the other journal have been redacted.

Reviewer Comments & Decisions:

Decision Letter, initial version:

Date:	29th April 21 03:21:39
From:	jie.wang@nature.com
To:	susan.gasser@fmi.ch
BCC:	ncb@springernature.com
Subject:	Decision on Nature Cell Biology submission NCB-G45398-T
Message:	Dear Dr Gasser,

Thank you for allowing Nature Cell Biology the opportunity to consider your manuscript, "H3K9me blocks transcription factor activity in differentiated cells to ensure tissue integrity", for publication. ***[REDACTED]*** and Nature Cell Biology are editorially independent. However, when authors transfer a manuscript to Nature Cell Biology after it has been reviewed for ***[REDACTED]***, ***[REDACTED]*** passes the referees' comments (and their identities) to us, with the author's permission.

I have now discussed the manuscript and the ***[REDACTED]*** referees' comments along with the rebuttal in detail with my colleagues, and we believe that your findings would be of interest to this journal's readership. However, the criticisms raised by the referees are substantial, and in our view would need to be addressed with considerable revisions before we can consider publication in Nature Cell Biology.

Nature Cell Biology editors discuss the referee reports in detail within the editorial team, including the chief editor, to identify key referee points that should be addressed with priority, and requests that are overruled as being beyond the scope of the current study. To guide the scope of the revisions, I have listed these points below. I should stress that the referees' concerns point to a premature dataset and these points would need to be addressed with experiments and data, and reconsideration of the study for this journal and re-engagement of referees would depend on strength of these revisions.

We are committed to providing a fair and constructive peer-review process, so please feel free to contact me if you would like to discuss any of the referee comments further.

In particular, it would be essential to:

a) strengthen the claim that the observed gene expression changes can be explained by transcription factor (TF) expression patterns, as noted by referee 2. We appreciate that you will profile the TF binding to DNA via ChIP-qPCR. We also appreciate the technical difficulty in performing ChIP-seq with e.elegans embryos. However, as suggested by referee 2, alternatively, the genome-wide TF association could be assessed by ChIC-seq, which has already been used to profile histone mark distribution in this study. If technically feasible, we encourage you to perform ChIC-seq, instead of ChIP-qPCR, to provide a genome-wide view of chromatin association of certain TFs in WT vs H3K9me3 mutants.

[REDACTED]

b) add the data related to nuclear envelope to boost the advance, as proposed in the rebuttal. In this regard, we wonder if the re-activation of group-A genes in the absence of gaining chromatin accessibility is due to their dissociation from the nuclear envelope in the H3K9me mutants. To address it, you may consider performing emerin Dam-ID analysis in H3K9me mutants to check whether group-A genes are indeed dissociated from the nuclear envelope. Alternatively, you may check whether genes upregulated in CEC-4 mutant (reported in ref.5) overlap with group-A genes.

c) All other referee concerns pertaining to strengthening existing data, providing controls, methodological details, clarifications and textual changes, should also be addressed.

d) Finally please pay close attention to our guidelines on statistical and methodological reporting (listed below) as failure to do so may delay the reconsideration of the revised manuscript. In particular please provide:

- a Supplementary Figure including unprocessed images of all gels/blots in the form of a multi-page pdf file. Please ensure that blots/gels are labeled and the sections presented in the figures are clearly indicated.

- a Supplementary Table including all numerical source data in Excel format, with data for different figures provided as different sheets within a single Excel file. The file should include source data giving rise to graphical representations and statistical descriptions in the paper and for all instances where the figures present representative experiments of multiple independent repeats, the source data of all repeats should be provided.

We would be happy to consider a revised manuscript that would satisfactorily address these points, unless a similar paper is published elsewhere, or is accepted for publication in Nature Cell Biology in the meantime.

When revising the manuscript please:

- ensure that it conforms to our format instructions and publication policies (see below and https://www.nature.com/nature/for-authors).

- provide a point-by-point rebuttal to the last round of full referee reports verbatim.

- provide the completed Editorial Policy Checklist (found here https://www.nature.com/documents/nr-editorial-policy-checklist.pdf), Reporting Summary (found here https://www.nature.com/documents/nr-reporting-summary.pdf). This is essential for reconsideration of the manuscript and these documents will be available to editors and referees in the event of peer review. For more information see http://www.nature.com/authors/policies/availability.html or contact me.

When submitting the revised version of your manuscript, please pay close attention to our href="https://www.nature.com/nature-research/editorial-policies/image-integrity">Digital Image Integrity Guidelines. and to the following points below:

-- that unprocessed scans are clearly labelled and match the gels and western blots presented in figures.
-- that control panels for gels and western blots are appropriately described as loading on sample processing controls
-- all images in the paper are checked for duplication of panels and for splicing of gel lanes.

Finally, please ensure that you retain unprocessed data and metadata files after publication, ideally archiving data in perpetuity, as these may be requested during the peer review and production process or after publication if any issues arise.

Nature Cell Biology is committed to improving transparency in authorship. As part of our efforts in this direction, we are now requesting that all authors identified as 'corresponding author' on published papers create and link their Open Researcher and Contributor Identifier (ORCID) with their account on the Manuscript Tracking System (MTS), prior to acceptance. ORCID helps the scientific community achieve unambiguous attribution of all scholarly contributions. You can create and link your ORCID from the home page of the MTS by clicking on 'Modify my Springer Nature account'. For more information please visit please visit www.springernature.com/orcid.

This journal strongly supports public availability of data. Please place the data used in your paper into a public data repository, or alternatively, present the data as Supplementary Information. If data can only be shared on request, please explain why in your Data Availability Statement, and also in the correspondence with your editor. Please note that for some data types, deposition in a public repository is mandatory - more information on our data deposition policies and available repositories appears below.

Please submit the revised manuscript files and the point-by-point rebuttal to the referee comments using this link:

[REDACTED]

*This url links to your confidential home page and associated information about manuscripts you may have submitted or be reviewing for us. If you wish to forward this email to co-authors, please delete the link to your homepage.

We would like to receive a revised submission within six months.

We hope that you will find our referees' comments and editorial guidance helpful. Please do not hesitate to contact me if there is anything you would like to discuss.

Best wishes,

Jie Wang

Jie Wang, PhD
Senior Editor
Nature Cell Biology

Tel: +44 (0) 207 843 4924
email: jie.wang@nature.com

Author Rebuttal to Initial comments

[REDACTED]

Decision Letter, first revision:

Date: 3rd August 21 00:34:29
From: jie.wang@nature.com
To: susan.gasser@fmi.ch
CC: ncb@springernature.com
Subject: Decision on Nature Cell Biology submission NCB-G45398A
Message:

*Please delete the link to your author homepage if you wish to forward this email to co-authors.

Dear Professor Gasser,

Your manuscript, "H3K9me blocks transcription factor activity in differentiated cells to ensure tissue integrity.", has now been seen by two of our original referees. As you will see from their comments (attached below) they still raised some important points that should be addressed before we can consider publication in Nature Cell Biology.

Nature Cell Biology editors discuss the referee reports in detail within the editorial team, including the chief editor, to identify key referee points that should be addressed with priority, and requests that are overruled as being beyond the scope of the current study. To guide the scope of the revisions, I have listed these points below. We are committed to providing a fair and constructive peer-review process, so please feel free to contact me if you would like to discuss any of the referee comments further.

In particular, it would be essential to:

a) address the persistent concerns regarding the relationship between TF presence and gene de-repression upon H3K9me3 loss, as noted by the two referees. Please also revise the title, result interpretation and discussion to avoid over-statement.

Referee 1:
The idea that TFs are re-used in different cell contexts, and that they play a key role in affecting the de-repression of gene expression when the chromatin context is altered is certainly a reasonable expectation. However, I'm not sure that the links are sufficiently well-drawn here. For instance, on P8 how do TFs relate to PGL-1 (representing germline) de-repression? Much regulation of germline gene expression seems to be dependent on 3' UTRs, chromatin state, and small RNA pathways, and much less so on specific TFs. See Merritt et al 2008 (Seydoux lab) as an example, but there are many reports consistent with this interpretation. Presumably, the pgl-1 promoter does not have binding sites for the somatic TFs. Does it? If so, which ones? In the discussion, the authors argue that at least a couple of somatic TFs (HLHs) also have a role in the germ line based on phenotype. I have some quibbles about whether these specific phenotypes can even be attributed to a germline function, but even if they are, a phenotype is many steps removed from showing actual dependence of specific genes on these TFs for gene expression. Finally, if I interpret fig 5f correctly, the correlation of gene expression with the presence of the corresponding motif is not great (2/4). How does this work? In sum, there are some interesting correlations, but I think that the interpretation of the data regarding TFs needs to be more cautious in the paper.

Referee 2:
2. In response to all my suggestions regarding the possibility that TF motifs or TF expression levels can explain why some genes are de-repressed and some are not, upon loss of K9me3, the authors respond that K9me3 regulates TF binding at only a subset of tissue-specific genes, and inversely, that the presence of a TF motif cannot solely explain derepression/lack of derepression phenotype. This is perhaps because they do not seem to be able to identify enriched motifs in unchanged genes to use as control and thus it is not clear whether the absence of the corresponding TFs can explain their failure to become activated. While I understand the complexity of the phenomena, and the analysis, put simply, the authors' data show that K9me3 loss leads to de-repression of a subset of genes BUT we do not fully know why. Their model with TF simply cannot explain all of it, and the authors must therefore significantly tone down their conclusions (and I presume the title itself as well).

3. In fact and along the same lines, the conclusions are a little overstated. Based on their Emerin-DamID analysis, the authors themselves acknowledge in their rebuttal that: "Together these results suggest that genes that lose their heterochromatic state upon loss of H3K9me respond to a complex interplay of subnuclear localization, TF recruitment and aberrant enhancer activity." In some cases, they can link to the TF binding, but in some others it seems to be rather the nuclear organisation state, which determines the outcome of gene expression upon loss of K9me3.

I think this point is important, as the title is not really supported in a so black/white manner. My request is to ask the authors to provide a revision with a more balanced title, discussion, and integration that their data suggest that other features than TF binding (and K9me3) explain changes in gene expression and/or accessibility (e.g. nuclear organisation).

b) expand the distance analysis to the repeat family level, as noted by referee 2:
1. In response to my comment on whether the authors can rule out that the changes in gene expression observed upon met/2 and/or set-25 deletion are NOT driven by changes in expression/regulatory function of neighbouring repeats, the authors performed a distance analysis, included in Extended Data Fig. 5a and conclude that they find no enrichment of repeats. However, this analysis only makes sense, if it is performed at the very least at the repeat family level, and not with all the repeats confounded. Given the diversity in the regulation of TE expression amongst individual families, a distance analysis with 'all' repeats is not adequate. The authors should expand the analysis in Extended Figure 5a accordingly, to the repeat family level at the very least.

c) validate the results for genes that behave like Group B genes in muscle with an alternative approach other than the heterochromatic reporter assay, as noted by referee 2:
4. Lastly, the authors use their heterochromatic reporter as a validation strategy for genes that behave like Group B genes in muscle. However, this reporter may not be representative for the gene behaviour, as it is highly repetitive. While I appreciate that this reporter has been extremely useful to identify factors and behaviours for screening based in imaging readouts, it may not be representative for specific, single copy genes. The authors should either use an alternative validation strategy (FISH for example?) and in any case, they should explicitly discuss with caution their data using this heterochromatic repeat.

d) All other referee concerns pertaining to providing controls, methodological details, clarifications and textual changes, should also be addressed.

e) Finally please pay close attention to our guidelines on statistical and methodological reporting (listed below) as failure to do so may delay the reconsideration of the revised manuscript. In particular please provide:

- a Supplementary Figure including unprocessed images of all gels/blots in the form of a multi-page pdf file. Please ensure that blots/gels are labeled and the sections presented in the figures are clearly indicated.

- a Supplementary Table including all numerical source data in Excel format, with data for different figures provided as different sheets within a single Excel file. The file should include source data giving

rise to graphical representations and statistical descriptions in the paper and for all instances where the figures present representative experiments of multiple independent repeats, the source data of all repeats should be provided.

We therefore invite you to take these points into account when revising the manuscript. In addition, when preparing the revision please:

- ensure that it conforms to our format instructions and publication policies (see below and https://www.nature.com/nature/for-authors).

- provide a point-by-point rebuttal to the full referee reports verbatim, as provided at the end of this letter.

- provide the completed Reporting Summary (found here https://www.nature.com/documents/nr-reporting-summary.pdf). This is essential for reconsideration of the manuscript and will be available to editors and referees in the event of peer review. For more information see http://www.nature.com/authors/policies/availability.html or contact me.

When submitting the revised version of your manuscript, please pay close attention to our href="https://www.nature.com/nature-research/editorial-policies/image-integrity">Digital Image Integrity Guidelines. and to the following points below:

-- that unprocessed scans are clearly labelled and match the gels and western blots presented in figures.
-- that control panels for gels and western blots are appropriately described as loading on sample processing controls
-- all images in the paper are checked for duplication of panels and for splicing of gel lanes.

Finally, please ensure that you retain unprocessed data and metadata files after publication, ideally archiving data in perpetuity, as these may be requested during the peer review and production process or after publication if any issues arise.

Nature Cell Biology is committed to improving transparency in authorship. As part of our efforts in this direction, we are now requesting that all authors identified as ‘corresponding author’ on published papers create and link their Open Researcher and Contributor Identifier (ORCID) with their account on the Manuscript Tracking System (MTS), prior to acceptance. ORCID helps the scientific community achieve unambiguous attribution of all scholarly contributions. You can create and link your ORCID from

the home page of the MTS by clicking on ‘Modify my Springer Nature account’. For more information please visit please visit www.springernature.com/orcid.

This journal strongly supports public availability of data. Please place the data used in your paper into a public data repository, or alternatively, present the data as Supplementary Information. If data can only be shared on request, please explain why in your Data Availability Statement, and also in the correspondence with your editor. Please note that for some data types, deposition in a public repository is mandatory - more information on our data deposition policies and available repositories appears below.

Please submit the revised manuscript files and the point-by-point rebuttal to the referee comments using this link:

[REDACTED]

*This url links to your confidential home page and associated information about manuscripts you may have submitted or be reviewing for us. If you wish to forward this email to co-authors, please delete the link to your homepage.

We would like to receive the revision within four weeks. If submitted within this time period, reconsideration of the revised manuscript will not be affected by related studies published elsewhere, or accepted for publication in Nature Cell Biology in the meantime. We would be happy to consider a revision even after this timeframe, but in that case we will consider the published literature at the time of resubmission when assessing the file.

We hope that you will find our referees' comments, and editorial guidance helpful. Please do not hesitate to contact me if there is anything you would like to discuss.

Best wishes,

Jie Wang

Jie Wang, PhD
Senior Editor
Nature Cell Biology

Tel: +44 (0) 207 843 4924

email: jie.wang@nature.com

Reviewers' Comments:

Reviewer #1:
Remarks to the Author:
This manuscript provides a very in-depth analysis of the relationships between heterochromatic state, chromatin accessibility, subnuclear localization and tissue-specific transcription factor activity to understand how these factors combine to affect gene de-repression. This is a monumental task, and as mentioned before, this body of work is very deep and carefully analyzed. Moreover, it has undergone substantial revision since the previous submission, and the authors have taken into account the suggestions by the reviewers. Overall, the manuscript is improved and in its current format will provide significant insight to the field. However, there is still one issue that seems problematic.

The idea that TFs are re-used in different cell contexts, and that they play a key role in affecting the de-repression of gene expression when the chromatin context is altered is certainly a reasonable expectation. However, I'm not sure that the links are sufficiently well-drawn here. For instance, on P8 how do TFs relate to PGL-1 (representing germline) de-repression? Much regulation of germline gene expression seems to be dependent on 3' UTRs, chromatin state, and small RNA pathways, and much less so on specific TFs. See Merritt et al 2008 (Seydoux lab) as an example, but there are many reports consistent with this interpretation. Presumably, the pgl-1 promoter does not have binding sites for the somatic TFs. Does it? If so, which ones? In the discussion, the authors argue that at least a couple of somatic TFs (HLHs) also have a role in the germ line based on phenotype. I have some quibbles about whether these specific phenotypes can even be attributed to a germline function, but even if they are, a phenotype is many steps removed from showing actual dependence of specific genes on these TFs for gene expression. Finally, if I interpret fig 5f correctly, the correlation of gene expression with the presence of the corresponding motif is not great (2/4). How does this work? In sum, there are some interesting correlations, but I think that the interpretation of the data regarding TFs needs to be more cautious in the paper.

Line 12, 33 – write out wild type and/or establish the abbreviation

Reviewer #2:
Remarks to the Author:

For the most part, the authors have addressed the comments that I raised in my original revision for ***[REDACTED]***. However, I do have some remaining points that the authors have not yet fully addressed and should be addressed, as follows:

1. In response to my comment on whether the authors can rule out that the changes in gene expression observed upon met/2 and/or set-25 deletion are NOT driven by changes in expression/regulatory function of neighbouring repeats, the authors performed a distance analysis, included in Extended Data Fig. 5a and conclude that they find no enrichment of repeats. However, this analysis only makes sense, if it is performed at the very least at the repeat family level, and not with all the repeats confounded. Given the diversity in the regulation of TE expression amongst individual families, a distance analysis with 'all' repeats is not adequate. The authors should expand the analysis in Extended Figure 5a accordingly, to the repeat family level at the very least.

2. In response to all my suggestions regarding the possibility that TF motifs or TF expression levels can explain why some genes are de-repressed and some are not, upon loss of K9me3, the authors respond that K9me3 regulates TF binding at only a subset of tissue-specific genes, and inversely, that the presence of a TF motif cannot solely explain derepression/lack of derepression phenotype. This is perhaps because they do not seem to be able to identify enriched motifs in unchanged genes to use as control and thus it is not clear whether the absence of the corresponding TFs can explain their failure to become activated. While I understand the complexity of the phenomena, and the analysis, put simply, the authors' data show that K9me3 loss leads to de-repression of a subset of genes BUT we do not fully know why. Their model with TF simply cannot explain all of it, and the authors must therefore significantly tone down their conclusions (and I presume the title itself as well).

3. In fact and along the same lines, the conclusions are a little overstated. Based on their Emerin-DamID analysis, the authors themselves acknowledge in their rebuttal that: "Together these results suggest that genes that lose their heterochromatic state upon loss of H3K9me respond to a complex interplay of subnuclear localization, TF recruitment and aberrant enhancer activity." In some cases, they can link to the TF binding, but in some others it seems to be rather the nuclear organisation state, which determines the outcome of gene expression upon loss of K9me3.
I think this point is important, as the title is not really supported in a so black/white manner. My request is to ask the authors to provide a revision with a more balanced title, discussion, and integration that their data suggest that other features than TF binding (and K9me3) explain changes in gene expression and/or accessibility (e.g. nuclear organisation).

4. Lastly, the authors use their heterochromatic reporter as a validation strategy for genes that behave like Group B genes in muscle. However, this reporter may not be representative for the gene behaviour, as it is highly repetitive. While I appreciate that this reporter has been extremely useful to identify factors and behaviours for screening based in imaging readouts, it may not be representative for

specific, single copy genes. The authors should either use an alternative validation strategy (FISH for example?) and in any case, they should explicitly discuss with caution their data using this heterochromatic repeat.

Author Rebuttal, first revision:

Reviewers' Comments:

Reviewer #1:
Remarks to the Author:
This manuscript provides a very in-depth analysis of the relationships between heterochromatic state, chromatin accessibility, subnuclear localization and tissue-specific transcription factor activity to understand how these factors combine to affect gene de-repression. This is a monumental task, and asmentioned before, this body of work is very deep and carefully analyzed. Moreover, it has undergone substantial revision since the previous submission, and the authors have taken into account the suggestions by the reviewers. Overall, the manuscript is improved and in its current format will providesignificant insight to the field.

However, there is still one issue that seems problematic. The idea that TFs are re-used in different cell contexts, and that they play a key role in affecting the de-repression of gene expression when the chromatin context is altered is certainly a reasonable expectation. However, I'm not sure that the links are sufficiently well-drawn here.

That H3K9me contributes to TF "re-use" in different tissues is indeed somewhat speculative, although it is a very reasonable *inference* or *implication* based on the data we present. We havecarefully revised the text and removed any claim that we prove "re-use". Rather we suggest in the discussion that "tissue-specific target restriction by H3K9me on both enhancers and promoters may allow TFs to promote distinct expression programs at different stages of development and/or in different tissues." The fact that TFs are used multiple times during development in multiple tissues is well established in the literature (notably for Onecut1/2, which is the CEH-48 homolog[1], but see also ref [2]). The data we present show that the depositionof H3K9me in differentiated tissues *restricts expression of different sets of genes in different tissues, and controls yet other TFs and genes in embryos.* Further arguments are as follows:

1. We show that in the absence of H3K9me there is aberrant gene activation in muscle and seamcells: about one third of the derepressed loci are in common but two thirds are different in the two tissues. Almost all are distinct from those derepressed in embryos.
2. The derepressed genes have motifs both for tissue-distinct *and* shared TFs , and aberrant gene activation depends on the presence of the TFs recognizing these motifs (Figure 5a,b for*pgl-1* and 5e for all derepressed genes). Very important !
3. H3K9me is shown to block the binding of at least one of the key TFs (by ChIP for CEH-48)

tothe promoter regions of H3K9me-sensitive genes (we find no binding unless the cells lack H3K9me: Figure 5f).
4. Since the relevant TFs are expressed in a range of tissues (Figure 4d), we believe it is reasonable to speculate - at least in the discussion - that tissue-specific H3K9me at promoters/ enhancers represses different subsets of genes in different tissues. In other words, since H3K9me can block TF activity, TFs can be "re-used" or "have an alternative use" in different tissues as long as the H3K9me HMTs are intact. We now also clearly state that the TFs may work*with* co-factors or chromatin modifiers, but note that remodelers/modifiers are rarely tissue- specific.

What would nail the "reuse" argument, of course, would be to show in multiple tissues, or at multiple stages of development, that *tissue-specific H3K9me patterns block TF binding in one tissue but not in another.* The tissue-specific ChIC-seq in worms (e.g. especially for TFs) is not yet amenable for use in sorted cells (representing different tissues). We have done the next best thing: we correlated H3K9me distribution *in muscle* with gene derepression *in muscle*, we identify the TFs involved, and show that whereas some of the relevant TFs are expressed only inone of the tissues (or stages of development), others are expressed in both muscle and seam cells, or in embryos *and* one or both tissues (Figure 4d). Moreover, we show that H3K9me can block TF binding (by ChIP). We have kept the "re-use" speculation for the discussion, but we think that our work goes further than any paper in the past, to show how H3K9me controls geneexpression in a tissue-specific manner. Note that we also show that the loss of H3K9me generates developmental defects (at least in muscle).

For instance, on P8 how do TFs relate to PGL-1 (representing germline) de-repression? Much regulationof germline gene expression seems to be dependent on 3' UTRs, chromatin state, and small RNA pathways, and much less so on specific TFs. See Merritt et al 2008 (Seydoux lab) as an example, but there are many reports consistent with this interpretation.

While some germline genes are regulated by a complex set of post-transcriptional mechanisms, there are specific transcription factors that are involved in regulating germline gene expression throughout evolution[3-7]. Further, Merrit et al 2008 show that while the 3`UTR of ~30 tested germline genes restricts the abundance of the corresponding proteins to specific stages of germcell maturation, the underlying germline-specific expression *is* regulated by the promoter sequence. In fact, germline specific promoter sequences (the *mex-5* promoter identified by Merrit et al 2008, for instance) are commonly used to drive germline specific reporters[8, 9]. We have now analyzed the TF motif enrichment in the derepressed germline genes in muscle and seam cells and we find the same TFs motifs (e.g. HLH-25/27/28/29, CEH-48…) as in the completelist of derepressed genes (included in Extended data Fig. 5e). <u>This is mentioned in the text with respect to the *pgl-1* expression figure (Fig. 5a,b</u>). Of course, the control is also included: when we analyse H3K9me-marked genes *not* derepressed by loss of H3K9me (whether germline or not), we do not find these TF motifs enriched (Extended data Fig. 5d).

Presumably, the pgl-1 promoter does not have binding sites for the somatic TFs. Does it? If so, whichones?

Pgl-1 was chosen as a readout for H3K9me sensitivity specifically because its promoter sequence contains motifs for CEH-48, HLH-25/-27, HLH-28/-29 and MADF-9, among others. We now provide a better description of the *pgl-1* promoter in the manuscript, along with Supplemental Table 3 that lists all TF motifs in the *pgl-1* promoter. Because knockdown of theseTFs by RNAi mitigates the aberrant expression of *pgl-1* observed in the *met-2 set-25* mutant, themost plausible explanation is that these TFs directly regulate *pgl-1* expression through their action as TFs. We confirm that *pgl-1* is upregulated at the mRNA level (not just the protein level)by RNA-seq in *met-2* deficient L1 larvae, and this derepression is dependent on the presence of CEH-48, HLH-25/-27 and partially on HLH-28/-29 (Extended data Fig. 6h). We also include proof that it bears H3K9me3 in muscle cells (Ext. data Fig. 2c)

In the discussion, the authors argue that at least a couple of somatic TFs (HLHs) also have a role in the germ line based on phenotype. I have some quibbles about whether these specific phenotypes can even be attributed to a germline function, but even if they are, a phenotype is many steps removed from showing actual dependence of specific genes on these TFs for gene expression.

We agree that the phenotypes resulting from loss of these TFs may not be directly related to germline gene expression. We now altered the text to read: "Finally, loss of the TFs HLH- 25/HLH-27 and HLH-28/HLH-29, which also drive gene derepression in the *met-2 set-25* mutant muscle, have pleiotropic defects in multiple tissues, including germline, in otherwise wt worms".There is very little literature on these HLH TFs, yet we show in Fig. 5 that they do regulate *pgl-1* expression in the absence of H3K9me; RNA-seq data for *pgl-1* is now in Extended data Fig. 6h.

Finally, if I interpret fig 5f correctly, the correlation of gene expression with the presence of thecorresponding motif is not great (2/4). How does this work?

Going back to the text, we saw that we did not label the figure well nor explain our controls sufficiently, which created some confusion regarding the ChIP data. In fact, for CEH-48::GFP (which is expressed in muscle and seam cells and has motifs enriched in the promoters of derepressed genes) 2 of 2 derepressed genes bearing perfect matches to its motif showed increased binding in the absence of H3K9me (100%). On top of this, one derepressed gene that did not contain a perfect motif also showed increased binding. We have gone back and checked that promoter for "near matches" to the CEH-48 motif. There are two near perfect matches, and seven motifs with 6 of 9 positions matching the consensus. This level of near match was not observed in our negative controls. Thus we now label the third positive promoter for CEH-48 ChIPas containing two near perfect motifs (Figure 5f), such that three of three H3K9me-sensitive promoters bearing CEH-48 motifs show that the TF cannot bind in wt, while it can in *met-2 set-25*worms. For the two *not derepressed* control genes lacking the motif, CEH-48 did not bind. Similarly, at a *derepressed* gene lacking the motif, CEH-48 did not bind. We have explained this clearly in the revised text.

It was perhaps not properly explained that TFs KLF-3 and END-3, which we used as negative TF- ChIP controls, are embryo-specific and therefore not expected to show binding at any promoter in these L3 larvae. Indeed, they do not, even though 2 of the 5 promoters tested contained KLF-3or END-3 motifs. In summary, we present negative controls for factors that are not present, for motifs that are not present, and for genes that are expressed but not affected by *met-2 set-25,* along with three positive promoter ChIP results. We hope the data are now clearly described as they are very important to the paper.

In sum, there are some interesting correlations, but I think that the interpretation of the data regarding TFs needs to be more cautious in the paper.

We have toned down our conclusions regarding “TF re-use”. Nonetheless, based on phenotypes observed and motifs mapped, the TFs necessary for *met-2 set-25* gene derepression do have the potential to be active in multiple tissues. Perhaps it helps that we now show that the *pgl-1* promoter contains motifs for TFs that regulate its expression in the absence of H3K9me; in wt worms *pgl-1* expression in muscle and pharynx is blocked in a *met-2 set-25* dependent manner, and H3K9me3 enrichment at the *pgl-1* locus is now added (Extended data Fig. 2c).

Line 12, 33 – write out wild type and/or establish the abbreviation done

Reviewer #2:
Remarks to the Author:
For the most part, the authors have addressed the comments that I raised in my original revision for ***[REDACTED]***. However, I do have some remaining points that the authors have not yet fully addressed andshould be addressed, as follows:

1. In response to my comment on whether the authors can rule out that the changes in gene expression observed upon met/2 and/or set-25 deletion are NOT driven by changes in expression/regulatory function of neighbouring repeats, the authors performed a distance analysis, included in Extended Data Fig. 5a and conclude that they find no enrichment of repeats. However, this analysis only makes sense, ifit is performed at the very least at the repeat family level, and not with all the repeats confounded. Given the diversity in the regulation of TE expression amongst individual families, a distance analysis with ‘all’ repeats is not adequate. The authors should expand the analysis in Extended Figure 5a accordingly, to the repeat family level at the very least.

We agree that the repeat-proximity analysis makes more sense when presented as individual repeat families. We have now included this in Extended data Fig. 5a,b, and cite it both in results and in the discussion. Most repeats were on average further away or similarly spaced from the TSS of the derepressed genes as from the TSS of a random set of expressed genes (same numberof genes). The DNA, Harbinger, Merlin, Chapeav and TcMar-Tc4 repeat families were on averagea little bit closer to the TSS of genes derepressed in the *met-2 set-25* mutant than to a random set of TSS. However, the

mean distance between the TSSs and repeats of the Harbinger, Merlin and TcMar-Tc4 repeat family was 3.7 Mb, 4.2 Mb and 154 kb, respectively, which on worm chromosomes is enormous, and thus cannot be relevant as a general control mechanism. The closest Repeat-TSS mean distance mapped among these elements was 3.9 kb for the DNA repeat, which is still quite distant for worms. In addition, as mentioned in the discussion, "only 87 (26%) of the 331 derepressed genes have any repeat element within 1kb of the corresponding TSS, whereas 34% of all genes in the genome have a repeat element < 1kb from their TSS. Thus, there is actually an underrepresentation of repeats near derepressed genes."

Although none of our genome-wide analyses argues for a function of repeat elements in the misregulation of genes upon loss of H3K9me, we agree with the reviewer that the repression of genes by proximal repeat elements seems to occur in other systems and is thus cannot be totallydismissed. For this reason, we now added the following statement to the discussion: "Nonetheless, given the well-described function of H3K9me at repetitive elements, we do not exclude that individual repeats may function as regulatory sequences for some misexpressed genes. Even if repeats were to nucleate and thus help target HMTs to genes, TF binding is still likely to be necessary for the derepression of *met-2 set-25* sensitive genes."

Indeed, in the few instances in which people have analyzed examples of repeat mediated gene regulation[10], repression was achieved by restricting the binding of specific TFs to regulatory sequences in the repeat element, which fits our model of TF exclusion perfectly. In any case, weinclude the analysis of individual repeat families now in Extended Fig. 5a,b.

2. In response to all my suggestions regarding the possibility that TF motifs or TF expression levels can explain why some genes are de-repressed and some are not, upon loss of K9me3, the authors respond that K9me3 regulates TF binding at only a subset of tissue-specific genes, and inversely, that the presence of a TF motif cannot solely explain derepression/lack of derepression phenotype. This is perhaps because they do not seem to be able to identify enriched motifs in unchanged genes to use as control and thus it is not clear whether the absence of the corresponding TFs can explain their failure tobecome activated. While I understand the complexity of the phenomena, and the analysis, put simply, the authors' data show that K9me3 loss leads to de-repression of a subset of genes BUT we do not fully know why. Their model with TF simply cannot explain all of it, and the authors must therefore significantly tone down their conclusions (and I presume the title itself as well).

While there may indeed be other parameters that affect TF binding or activity, we show very clearly that among the set of H3K9me-regulated genes, there is a requirement for the relevantTFs for derepression in a *met-2 set-25* mutant. As explained above to rev. 1, the logic is this:

1. The derepressed genes respond to loss of H3K9me, differently in two different tissues. 2. Thegene promoters are enriched for a subset of TF motifs. Naturally this does not mean that these are the only TFs involved nor that every site at which these TFs bind, bears H3K9me in wt. 3. Nonetheless, the loss of the relevant TF by RNAi prevents derepression upon loss of

H3K9me. This is pretty compelling evidence that TFs are needed to drive the derepression of genes that lose H3K9me. Finally, and most importantly: 4. we show that the binding of at least one of the relevant TFs (CEH-48) to the promoters of misregulated genes, occurs only if H3K9me is lost (and is motif dependent). From this we conclude that TF binding can be regulated by H3K9me and that TF binding is required for gene derepression upon loss of H3K9me.

Of course, our list of relevant TFs is not exhaustive – we only identify those that were statistically enriched in the promoters of the affected genes. Undoubtedly many ubiquitously expressed factors also bind these promoters, and cooperate with factors like CEH-48. We statethis clearly in the text (see above). Nonetheless, loss of CEH-48 (and HLH-25/27, as well as others) are needed for the transcription of promoters that lose H3K9me (Figure 5).

In general, we feel that this reviewer wants us to explain why the *other* 87% of H3K9me-markedgenes are *not* derepressed upon loss of H3K9me in muscle. Obviously there are other repressivemarks, and some coincide with H3K9me – the one we know something about is H3K27me3, andthis mark overlaps extensively with H3K9me in worms. But there are other mechanisms as well, including Argonaut mediated mRNA degradation, which in *C elegans* works not only in germline but also in somatic cells (shown for worm embryos in Padeken et al G&D 2021). Our goal was not to identify all existing repression mechanisms, but instead to ask*, Is H3K9me responsible for tissue-specific gene repression, and if so, how? Does it block TF binding, chromatin opening, subnuclear position or enhancer control? Or, all of the above?* Our question was not "Is H3K9me*the unique* gene silencing mechanism in worms?" The answer to the latter is obviously no; if it were yes, *met-2 set-25* embryos would not develop into adults (*met-2 nrde-3* mutants are largely embryonic lethal). We do discuss various parameters that influence TF function, including interactions with other chromatin modifying complexes. It is clear that in the *met-2 set-25* mutant, the TFs we identified bind and activate but a subset of the complement of genesand loci that lose H3K9me in the *met-2 set-25* mutant.

Please note that in Extended data Fig. 5d (formerly c), we already included the analysis showing that the genes that are enriched for H3K9me3 but *do not* change expression upon loss of H3K9me, have no specific TF enrichment patterns. That does not mean they do not bind TFs, butcollectively they bind a *diversity* of TFs, so that none is statistically enriched. TF motif enrichment is a tool to identify relevant TFs, so that we can test whether or not they are necessary for gene derepression upon loss of H3K9me. RNAi was then used to antagonize these TFs and it was shown to mitigate *met-2 set-25* provoked gene derepression, both on an organismal level by RNA-seq (Fig. 5e) and the single cell level by microscopy (Fig. 5a,b).

Overall, this seems to us to be compelling evidence that TFs are essential drivers of *met-2 set-25* dependent gene derepression, which is what our title states. While not totally surprising, this was never shown in the context of organismal development. What *was* surprising was the fact that there are many more sites that become ATAC-seq sensitive upon loss of H3K9me, than genes derepressed,

and that there is no obligate correlation of ATAC-seq sensitivity with expression. We stress in our discussion that a subset of the TFs responsible for derepression, appear to drive chromatin decompaction coordinately (Class B genes), while another set of TFs that binds enhancers drives ATAC-seq sensitivity without derepression (Class C). These latter probably work by interacting with additional chromatin modifying complexes (e.g. SWI/SNF, or CBP/p300), as we see enhanced H3K27ac (in response to loss of H3K9me) on enhancer elementsand me-sensitive intergenic elements. Intriguingly, however, H3K27ac acquisition did not occur at all genes derepressed by loss of H3K9me.

We regret that the reviewer finds this complicated, but these details should not obscure the very simple main message of the paper: TFs shared among tissues drive different sets of gene derepression events in different tissues, upon the loss of H3K9me. And, H3K9me can block TF activity. We do include the ATAC-seq data as it allows us to identify open promoters and enhancers; and we hear that other labs are soon reporting on similar disconnects between ATAC-sensitivity and gene derepression.

3. In fact and along the same lines, the conclusions are a little overstated. Based on their Emerin-DamIDanalysis, the authors themselves acknowledge in their rebuttal that: “Together these results suggest that genes that lose their heterochromatic state upon loss of H3K9me respond to a complex interplay ofsubnuclear localization, TF recruitment and aberrant enhancer activity.” In some cases, they can link to the TF binding, but in some others it seems to be rather the nuclear organisation state, which determines the outcome of gene expression upon loss of K9me3.
I think this point is important, as the title is not really supported in a so black/white manner. My requestis to ask the authors to provide a revision with a more balanced title, discussion, and integration that their data suggest that other features than TF binding (and K9me3) explain changes in gene expression and/or accessibility (e.g. nuclear organisation).

We have streamlined the discussion to spell out the logic of our conclusions. What we learned from our correlation of ATAC-seq, RNA-seq and Emerin-DamID data in muscle cells, is that tissuespecific *gene derepression* affects genes independently of their association with the nuclear periphery (both group A and B genes are derepressed, Group A is enriched near the periphery, Group B are not). Indeed, gene derepression depends on TF binding, and this can happen at the periphery or internally. Moreover, genes can be internal and silenced (i.e., the loss of peripheral anchoring (e.g. loss of *cec-4)* is not sufficient to alter expression[11]). What the muscle specific Emerin-DamID brought to our study is an ability to differentiate between derepressed genes that *gain ATAC accessibility* (Group B) from those that do not (Group A). Those showing a *gain inATAC sensitivity* along with their gene expression, *are more internally localized even when repressed*. This does not mean that position drives gene expression – rather it argues that the *derepressed chromatin status* may be different depending on whether a gene is at the nuclear envelope or in the nuclear interior.

Another insight, that goes beyond the role of TFs in gene activation, is that enhancer elements can become accessible upon loss of H3K9me, yet do not correlate with increased transcription of the

nearest gene (Figure 7). Enhancer elements, like promoters, likely require the binding of sequence specific TFs for the observed changes in accessibility (e.g. in *C. elegans* EOR-1, ZTF-3 and DAF-12/NHR-48). Exactly why they do not turn on adjacent genes is unclear, but it could well be that promoter binding TFs are missing. Understanding this will be another study, as it requires the mapping of enhancer-promoter interactions in a tissue-specific manner. We try notto overstate the impact of our observation that Group C loci correlate with putative enhancers, although this is highly interesting.

Despite all these additional observations, the key message is that which is in the title: a gain-of-function for specific TFs is responsible for expression changes at genes that respond to the loss of H3K9me in differentiated tissues.

4. Lastly, the authors use their heterochromatic reporter as a validation strategy for genes that behave like Group B genes in muscle. However, this reporter may not be representative for the gene behaviour,as it is highly repetitive. While I appreciate that this reporter has been extremely useful to identify factors and behaviours for screening based in imaging readouts, it may not be representative for specific, single copy genes. The authors should either use an alternative validation strategy (FISH for example?) and in any case, they should explicitly discuss with caution their data using this heterochromatic repeat.

We understand the reviewers hesitation about the repetitive nature of the reporter potentially affecting its behavior; indeed we had highlighted this in the text. Nonetheless, we find it a useful example, as we can use the Emerin-DamID data (correlated with RNA-seq and ATAC-seq) for genome-wide validation of the results obtained with the reporter. The two data sets agree very well (both are in Figure 6), in that decompaction (or increased accessibility) in the absence of H3K9me is associated with being detached from the nuclear periphery in wild-type cells – both for the reporter and for endogenous sequences based on genome-wide DamID. We note that Meister et al. 2010 previously demonstrated that the nuclear positioning and expression of this heterochromatic reporter in somatic tissues is highly dependent on the specific promoter sequences contained in the integrated arrays[12]. Thus, if the repetitive nature of the reporter overrode other control mechanisms, we would not expect to monitor a tissue specific effect on derepression, accessibility or nuclear organization, since the reporter is repetitive in all tissues.

References of interest:

1. Kropp, P.A. & Gannon, M. Onecut transcription factors in development and disease. *Trends DevBiol* **9**, 43-57 (2016).
2. Briggs, J.A. *et al.* The dynamics of gene expression in vertebrate embryogenesis at single-cellresolution. *Science* **360**, eaar5780 (2018).
3. Wang, Z., Liu, C.-Y., Zhao, Y. & Dean, J. FIGLA, LHX8 and SOHLH1 transcription factor networks regulate mouse oocyte growth and differentiation. *Nucleic Acids Research* **48**, 3525-3541 (2020).
4. Magnúsdóttir, E. *et al.* A tripartite transcription factor network regulates primordial germ cellspecification in mice. *Nature Cell Biology* **15**, 905-915 (2013).

5. Nakaki, F. *et al.* Induction of mouse germ-cell fate by transcription factors in vitro. *Nature* **501**,222-226 (2013).
6. Nakamura, S. *et al.* A truncated form of a transcription factor Mamo activates vasa in Drosophilaembryos. *Communications Biology* **2**, 422 (2019).
7. Sano, H., Nakamura, A. & Kobayashi, S. Identification of a transcriptional regulatory region forgermline-specific expression of vasa gene in Drosophila melanogaster. *Mechanisms of Development* **112**, 129-139 (2002).
8. Zeiser, E., Frøkjær-Jensen, C., Jorgensen, E. & Ahringer, J. MosSCI and Gateway CompatiblePlasmid Toolkit for Constitutive and Inducible Expression of Transgenes in the C. elegans Germline. *PLOS ONE* **6**, e20082 (2011).
9. Serizay, J. *et al.* Distinctive regulatory architectures of germline-active and somatic genes in C.elegans. *Genome Research* **30**, 1752-1765 (2020).
10. Grow, E.J. *et al.* Intrinsic retroviral reactivation in human preimplantation embryos andpluripotent cells. *Nature* **522**, 221-225 (2015).
11. Gonzalez-Sandoval, A. *et al.* Perinuclear Anchoring of H3K9-Methylated Chromatin StabilizesInduced Cell Fate in C. elegans Embryos. *Cell* **163**, 1333-1347 (2015).
12. Meister, P., Towbin, B.D., Pike, B.L., Ponti, A. & Gasser, S.M. The spatial dynamics of tissue-specific promoters during C. elegans development. *Genes Dev* **24**, 766-782 (2010).

Decision Letter, second revision:

Date: 23rd August 21 22:08:52
From: jie.wang@nature.com
To: susan.gasser@fmi.ch
CC: ncb@springernature.com; ziqian.li@nature.com
Subject: Your manuscript, NCB-G45398B
Message:

Our ref: NCB-G45398B

23rd August 2021

Dear Dr. Gasser,

Thank you for submitting your revised manuscript "H3K9me selectively blocks transcription factor activity and ensures differentiated tissue integrity" (NCB-G45398B). It has now been seen by the original referees and their comments are below. The reviewers find that the paper has improved in revision, and

therefore we'll be happy in principle to publish it in Nature Cell Biology, pending minor revisions to comply with our editorial and formatting guidelines.

We are now performing detailed checks on your paper and will send you a checklist detailing our editorial and formatting requirements in about a week. Please do not upload the final materials and make any revisions until you receive this additional information from us.

Thank you again for your interest in Nature Cell Biology. Please do not hesitate to contact me if you have any questions.

Sincerely,

Jie Wang, PhD
Senior Editor
Nature Cell Biology

Tel: +44 (0) 207 843 4924
email: jie.wang@nature.com

Reviewer #1 (Remarks to the Author):

The last round of changes to the manuscript address my concerns satisfactorily.

Reviewer #2 (Remarks to the Author):

The authors have addressed most, although not all the points I raised. However, their improved discussion incorporates many of my points and therefore I can provide a positive recommendation for publication of this manuscript in NCB.

Decision letter, final requests:

Date:	30th August 21 23:59:40
From:	ziqian.li@nature.com
To:	susan.gasser@fmi.ch
CC:	ncb@springernature.com,jie.wang@nature.com
BCC:	ziqian.li@nature.com
Subject:	NCB: Your manuscript, NCB-G45398B
Message:	

natureresearch

Our ref: NCB-G45398B

30th August 2021

Dear Dr. Gasser,

Thank you for your patience as we've prepared the guidelines for final submission of your Nature Cell Biology manuscript, "H3K9me selectively blocks transcription factor activity and ensures differentiated tissue integrity" (NCB-G45398B). Please carefully follow the step-by-step instructions provided in the attached file, and add a response in each row of the table to indicate the changes that you have made. Ensuring that each point is addressed will help to ensure that your revised manuscript can be swiftly handed over to our production team.

We would like to start working on your revised paper, with all of the requested files and forms, as soon as possible (preferably within two weeks). Please get in contact with us if you anticipate delays.

When you upload your final materials, please include a point-by-point response to any remaining reviewer comments.

If you have not done so already, please alert us to any related manuscripts from your group that are under consideration or in press at other journals, or are being written up for submission to other journals (see: https://www.nature.com/nature-research/editorial-policies/plagiarism#policy-on-duplicate-publication for details).

In recognition of the time and expertise our reviewers provide to Nature Cell Biology's editorial process, we would like to formally acknowledge their contribution to the external peer review of your manuscript entitled "H3K9me selectively blocks transcription factor activity and ensures differentiated tissue integrity". For those reviewers who give their assent, we will be publishing their names alongside the published article.

Nature Cell Biology offers a Transparent Peer Review option for new original research manuscripts submitted after December 1st, 2019. As part of this initiative, we encourage our authors to support increased transparency into the peer review process by agreeing to have the reviewer comments, author rebuttal letters, and editorial decision letters published as a Supplementary item. When you submit your final files please clearly state in your cover letter whether or not you would like to participate in this initiative. Please note that failure to state your preference will result in delays in accepting your manuscript for publication.

Cover suggestions

As you prepare your final files we encourage you to consider whether you have any images or illustrations that may be appropriate for use on the cover of Nature Cell Biology.

Covers should be both aesthetically appealing and scientifically relevant, and should be supplied at the best quality available. Due to the prominence of these images, we do not generally select images featuring faces, children, text, graphs, schematic drawings, or collages on our covers.

We accept TIFF, JPEG, PNG or PSD file formats (a layered PSD file would be ideal), and the image should be at least 300ppi resolution (preferably 600-1200 ppi), in CMYK colour mode.

If your image is selected, we may also use it on the journal website as a banner image, and may need to make artistic alterations to fit our journal style.

Please submit your suggestions, clearly labeled, along with your final files. We'll be in touch if more information is needed.

Nature Cell Biology has now transitioned to a unified Rights Collection system which will allow our Author Services team to quickly and easily collect the rights and permissions required to publish your work. Approximately 10 days after your paper is formally accepted, you will receive an email in providing you with a link to complete the grant of rights. If your paper is eligible for Open Access, our Author Services team will also be in touch regarding any additional information that may be required to arrange payment for your article.

Please note that Nature Cell Biology is a Transformative Journal (TJ). Authors may publish their research with us through the traditional subscription access route or make their paper immediately open access through payment of an article-processing charge (APC). Authors will not be required to make a final decision about access to their article until it has been accepted. Find out more about Transformative Journals

Authors may need to take specific actions to achieve compliance with funder and institutional open access mandates. For submissions from January 2021, if your research is supported by a funder that requires immediate open access (e.g. according to Plan S principles) then you should select the gold OA route, and we will direct you to the compliant route where possible. For authors selecting the subscription publication route our standard licensing terms will need to be accepted, including our self-archiving policies. Those standard licensing terms will supersede any other terms that the author or any third party may assert apply to any version of the manuscript.

Please note that you will not receive your proofs until the publishing agreement has been received through our system.

For information regarding our different publishing models please see our Transformative Journals page. If you have any questions about costs, Open Access requirements, or our legal forms, please contact ASJournals@springernature.com.

Please use the following link for uploading these materials:
[REDACTED]

If you have any further questions, please feel free to contact me.

Best regards,

Ziqian Li
Editorial Assistant
Nature Cell Biology

On behalf of

Jie Wang, PhD
Senior Editor
Nature Cell Biology

Tel: +44 (0) 207 843 4924
email: jie.wang@nature.com

Reviewer #1:
Remarks to the Author:
The last round of changes to the manuscript address my concerns satisfactorily.

Reviewer #2:
Remarks to the Author:

The authors have addressed most, although not all the points I raised. However, their improved discussion incorporates many of my points and therefore I can provide a positive recommendation for publication of this manuscript in NCB.

Final Decision Letter:

Date:	17th September 21 04:52:19
From:	jie.wang@nature.com
To:	susan.gasser@fmi.ch
CC:	ncb@springernature.com
BCC:	rjsproduction@springernature.com;rjsart@springernature.com
Subject:	Decision on Nature Cell Biology submission NCB-G45398C
Message:	

Dear Dr Gasser,

I am pleased to inform you that your manuscript, "H3K9me selectively blocks transcription factor activity and ensures differentiated tissue integrity", has now been accepted for publication in Nature Cell Biology.

Thank you for sending us the final manuscript files to be processed for print and online production, and for returning the manuscript checklists and other forms. Your manuscript will now be passed to our production team who will be in contact with you if there are any questions with the production quality of supplied figures and text.

In approximately 10 business days you will receive an email with a link to choose the appropriate publishing options for your paper and our Author Services team will be in touch regarding any additional information that may be required.

You will not receive your proofs until the publishing agreement has been received through our system.

If you have any questions about our publishing options, costs, Open Access requirements, or our legal forms, please contact ASJournals@springernature.com

Before the manuscript is sent to our printers, we will make changes in the text that may be necessary either to make it conform with house style or to make it intelligible to our wide readership. We look particularly carefully at the titles of all papers to ensure that indexing will be accurate and that they are not unreasonably long. We will ask your approval before the copy is finalized, and you will soon receive

the edited proofs. Please check the text and figures carefully. Once your manuscript is typeset and you have completed the appropriate grant of rights, you will receive a link to your electronic proof via email with a request to make any corrections within 48 hours. If, when you receive your proof, you cannot meet this deadline, please inform us at rjsproduction@springernature.com immediately.

Once your paper has been scheduled for online publication, the Nature press office will be in touch to confirm the details. An online order form for reprints of your paper is available at https://www.nature.com/reprints/author-reprints.html. All co-authors, authors' institutions and authors' funding agencies can order reprints using the form appropriate to their geographical region.

Publication is conditional on the manuscript not being published elsewhere and on there being no announcement of this work to any media outlet until the online publication date in Nature Cell Biology.

Please note that Nature Cell Biology is a Transformative Journal (TJ). Authors may publish their research with us through the traditional subscription access route or make their paper immediately open access through payment of an article-processing charge (APC). Authors will not be required to make a final decision about access to their article until it has been accepted. Find out more about Transformative Journals

Authors may need to take specific actions to achieve compliance with funder and institutional open access mandates. For submissions from January 2021, if your research is supported by a funder that requires immediate open access (e.g. according to Plan S principles) then you should select the gold OA route, and we will direct you to the compliant route where possible. For authors selecting the subscription publication route our standard licensing terms will need to be accepted, including our self-archiving policies. Those standard licensing terms will supersede any other terms that the author or any third party may assert apply to any version of the manuscript.

To assist our authors in disseminating their research to the broader community, our SharedIt initiative provides you with a unique shareable link that will allow anyone (with or without a subscription) to read the published article. Recipients of the link with a subscription will also be able to download and print the PDF.

If your paper includes color figures, please be aware that in order to help cover some of the additional cost of four-color reproduction, Nature Research charges our authors a fee for the printing of their color figures. Please contact our offices for exact pricing and details.

As soon as your article is published, you will receive an automated email with your shareable link.

If you have not already done so, we strongly recommend that you upload the step-by-step protocols used in this manuscript to the Protocol Exchange (www.nature.com/protocolexchange), an open online resource established by Nature Protocols that allows researchers to share their detailed experimental know-how. All uploaded protocols are made freely available, assigned DOIs for ease of citation and are fully searchable through nature.com. Protocols and the Nature and Nature research journal papers in which they are used can be linked to one another, and this link is clearly and prominently visible in the online versions of both papers. Authors who performed the specific experiments can act as primary authors for the Protocol as they will be best placed to share the methodology details, but the Corresponding Author of the present research paper should be included as one of the authors. By uploading your Protocols to Protocol Exchange, you are enabling researchers to more readily reproduce or adapt the methodology you use, as well as increasing the visibility of your protocols and papers. You can also establish a dedicated page to collect your lab Protocols. Further information can be found at www.nature.com/protocolexchange/about

You can use a single sign-on for all your accounts, view the status of all your manuscript submissions and reviews, access usage statistics for your published articles and download a record of your refereeing activity for the Nature journals.

Please feel free to contact us if you have any questions.

With kind regards,

Jie Wang, PhD
Senior Editor
Nature Cell Biology

Tel: +44 (0) 207 843 4924
email: jie.wang@nature.com
